# Decoding the Integrated Stress Response of Pancreatic Cancer: Identifying a Serine‐dependent Tumor Subset Under Metabolic Relationships With CAFs.

**DOI:** 10.1002/advs.202515740

**Published:** 2026-02-17

**Authors:** Sauyeun Shin, Mehdi Liauzun, Jacobo Solorzano, Morgane Le Bras, Christine Jean, Benjamin Fourneaux, Margaux Dore, Lea Fevrier, Ismahane Belhabib, Alexia Brunel, Cindy Neuzillet, Marion Larroque, Carine Joffre, Stephane Rocchi, Nicolas Fraunhoffer, Aurelie Perraud, Muriel Mathonnet, Vera Pancaldi, Laetitia Linares, Juan Iovanna, Nelson Dusetti, Ola Larsson, Remy Nicolle, Stephane Pyronnet, Corinne Bousquet, Yvan Martineau

**Affiliations:** ^1^ Cancer Research Center of Toulouse (CRCT) INSERM U1037 ERL5294 CNRS University of Toulouse Toulouse France; ^2^ Institut de Recherche en Cancérologie de Montpellier (IRCM) INSERM U1194 ICM Université De Montpellier Montpellier France; ^3^ Medical Oncology Department Curie Institute Versailles Saint‐Quentin University Saint‐Cloud France; ^4^ Centre Méditerranéen de Médecine Moléculaire INSERM U1065 Université Cote d'Azur Nice France; ^5^ Centre de Recherche en Cancérologie de Marseille (CRCM) INSERM U1068 CNRS UMR 7258 Aix‐Marseille Université and Institut Paoli‐Calmettes Marseille France; ^6^ Department of Digestive Surgery Faculty of Medicine University Hospital of Limoges and INSERM U1308 – CAPTuR University of Limoges Limoges France; ^7^ Department of Oncology‐Pathology Karolinska Institutet Stockholm Sweden; ^8^ Centre de Recherche sur l'Inflammation (CRI) INSERM U1149 CNRS ERL 8252 Université Paris Cité Paris France; ^9^ Laboratoire d'excellence LABEX TOUCAN Toulouse France; ^10^ Equipe Labellisée Ligue Contre le Cancer

**Keywords:** cancer‐associated fibroblast, integrated stress response, mRNA translation, pancreatic cancer, serine metabolism

## Abstract

Pancreatic ductal adenocarcinoma (PDA) transcriptomic profiling has identified prognostic subtypes, yet patient‐specific first‐line therapies remain elusive. Here, we stratified PDA tumors by mRNA translation rates, a frequently dysregulated step in gene expression, using translatome profiling of 27 patient‐derived xenografts (PDXs). Unsupervised analysis revealed a distinct tumor subset with low global protein synthesis but sustained translation of Integrated Stress Response (ISR) mRNAs, including ATF4. These ISR‐activated cancer cells exhibited broad chemoresistance and apoptosis resistance, yet were auxotrophic for serine due to loss of PHGDH and CBS expression, impairing serine and cysteine biosynthesis. This vulnerability correlated with improved overall survival in patients with low expression of both enzymes. Notably, cancer‐associated fibroblasts (CAFs) reprogrammed by ISR‐activated cells, shifting from myCAF to iCAF phenotype with reduced collagen synthesis and glycine‐to‐serine conversion, produced serine and sustained tumor growth in amino acid‐depleted environments. Our findings demonstrate the power of translatome profiling to reveal stable, drug‐resistant PDA cell states and identify a targetable CAF‐tumor metabolic symbiosis, opening new avenues for therapeutic intervention in this highly lethal malignancy.

## Graphical abstract

Researchers identified a pancreatic cancer subset with low global protein synthesis but heightened translation of stress‐response genes like ATF4, rendering cells resistant to chemotherapy and apoptosis. These tumors depend on serine and cysteine from surrounding cancer‐associated fibroblasts (CAFs), which shift to produce serine by curbing collagen synthesis. Low levels of serine‐synthesizing enzymes PHGDH and CBS in such tumors predict better patient survival.

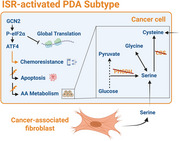

## 1 Introduction

Pancreatic ductal adenocarcinoma (PDA) is the fourth leading cause of cancer‐related death in industrialized countries, with a 5‐year survival rate below 10% [1]. This poor prognosis is largely due to late diagnosis, often at metastatic stages, and the limited response of patients to first‐line chemotherapies, Gemcitabine/Abraxane and FOLFIRINOX (35% and 32% response rates, respectively) [2]. “Omics” studies have contributed to a better characterization of PDA biology [3] and to patient stratification [4]. For example, exome sequencing has identified mutations in DNA repair genes in a subset of patients (11%), explaining their increased sensitivity to platinum‐based chemotherapies [5]. Moreover, all transcriptomic‐based classifications of PDA tumors converge on the existence of two major molecular subtypes (basal‐like and classical) and three stromal subtypes, which are prognostic but not predictive of response to specific chemotherapies. The involvement of cancer‐associated fibroblasts (CAFs) in stromal subtyping is underscored by their abundance and by their described metabolic interactions with tumor cells [6, 7].

PDA cancer cells possess intrinsic mechanisms that enable survival under stressful conditions, including the hypoxic and nutrient‐deprived tumor microenvironment. Adaptation to stress involves tightly regulated changes in both mRNA translation and DNA transcription. Notably, before transcriptional responses are initiated, cells typically downregulate global protein synthesis, a highly energy‐demanding process, allowing them to conserve resources and survive under adverse conditions [8]. This highlights the pivotal role of the protein synthesis machinery in PDA, which is often dysregulated to support tumor growth and persistence [9]. Moreover, alterations in translational regulators and transient activation of signaling pathways controlling protein synthesis have been proposed to facilitate adaptation to environmental changes in PDA [10, 11].

Regulation of protein synthesis can be quantitatively assessed by measuring translational efficiency (TE), defined as the fraction of mRNA copies from a given gene that are actively translated. Since protein levels often do not correlate directly with transcript abundance [12], partly due to transcript‐specific modulation of TE, this parameter provides unique biological insight. A well‐known example is the enhanced translation of activating transcription factor 4 (ATF4) mRNA during activation of the Integrated Stress Response (ISR), even while global translation is repressed. Consequently, ATF4 protein abundance is poorly correlated with its transcript levels [8]. ATF4 is a transcription factor essential for adaptation to diverse stresses, including oxidative stress, amino acid deprivation, endoplasmic reticulum (ER) protein misfolding, and chemotherapy exposure [8]. We therefore hypothesized that translatome profiling, rather than transcriptome analysis alone, could reveal additional layers of biological variability in PDA and refine patient stratification.

To test this, we performed a transcriptome‐wide analysis of highly translated mRNAs (associated with >3 ribosomes; the “translatome”) in 27 pancreatic patient‐derived xenografts (PDX). This approach identified a novel PDA subgroup characterized by a reduced global protein synthesis rate, but high TE for a subset of mRNAs involved in the ISR, including ATF4. Using primary PDX‐derived cell cultures and established PDA cell lines, we demonstrate that this “ISR‐activated” phenotype is marked by resistance to apoptosis and dependence on exogenous amino acids. Finally, we show that this metabolic vulnerability is mitigated by serine‐producing CAFs, revealing a critical tumor–stroma metabolic crosstalk that sustains the ISR‐activated phenotype.

## 2 Results

### 2.1 Translatome‐Based PDA Subtyping

**FIGURE 1:**
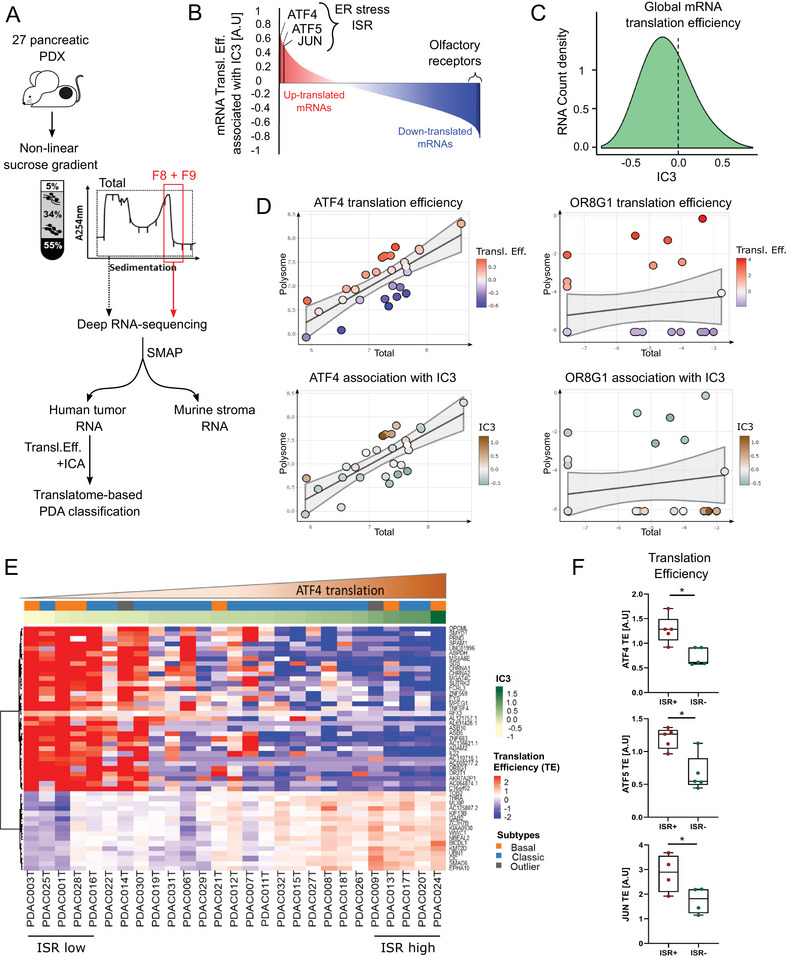
Translatome‐based PDA subtyping (A) Schematic of the translatome‐based PDA classification. SMAP was used to separate human and murine RNA sequences from total and polysomal fraction (F8/F9). Independent component analysis (ICA) has identified translational signatures. (B) Representation of mRNA Translation Efficiency associated with IC3. (C) Density of polysome RNA count associated with the component IC3 (Spearman correlation). (D) Scatter plot representing polysomal and total mRNA abundance in each PDX. Translation efficiency (TE) and association with IC3 are shown. (E) Heatmap representing the top correlated gene TE of PDX tumors associated with IC3. Basal‐like and classical classification of PDX and ATF4 expression are indicated. (F) The indicated mRNA TE have been plotted using the top 5 PDX correlated (ISR‐high) or not correlated (ISR‐low) with IC3. Data are presented as mean ± SD. P‐values were calculated using unpaired t‐test.

To define whether quantification of transcript‐selective TE allows identification of novel PDA tumor subtypes, we measured levels of translated mRNA in a collection of 27 PDX, also previously used to classify PDA tumors through transcriptomics [13]. PDX are considered as the most reliable avatars of individual patient tumors, as they recapitulate the histology and the molecular heterogeneities of the primary tumor [14]. PDX are hybrid models wherein human cancer cells proliferate and remodel the murine stroma, despite only partial compatibility in paracrine signaling between human and mouse cells. Using the SMAP algorithm [13], RNA sequencing reads originating from the human cancer cells can be separated from the murine microenvironment ones. To study total and efficiently translated mRNA (i.e., associated with >3 ribosomes), we used polysome‐profiling, coupled to standard RNAseq generating long reads (Figure 1A; Figure S1A). Then, estimated TEs were calculated for each transcript from human cancer cells in each tumor, using Anota2seq‐based approaches [15]. To carry out unsupervised assessment of TE across all tumors, we used Independent Component Analysis (ICA), (Figure 1A) and identified independent component 3 (IC3).

IC3 was correlated with an increased TE of several prototypical ER stress‐induced mRNAs, including ATF4, JUN and ATF5 (Figure 1B; Figure S1B), while low TE was observed for mRNAs encoding olfactory receptors (olfactory transduction KEGG pathway; NES: −2.52, *p*‐value: 9.9e‐03). The olfactory receptor multi‐gene family (about 800 genes) is over‐represented in the genome, suggesting that IC3 recapitulates a global attenuation of mRNA translation (Figure 1B), in line with a globally decreased density of polysome mRNA counts (Figure 1C). Moreover, we found high association between the IC3 component and the increased TE of ATF4 mRNA (Figure 1D), but not of olfactory receptor OR8G1 mRNA, one of the most translationally suppressed genes in IC3 (Figure 1D).

ISR reduces cap‐dependent translation initiation, via the phosphorylation of the eIF2α subunit leading to a decreased ternary complex formation, which selectively activates translation of mRNAs such as ATF4, ATF5, or JUN [16]. Consistently, the majority of IC3 top‐correlated transcripts showed a low TE and translational activation of a small subset of mRNAs (Figure 1E, TE in blue and red). Critically, IC3 does not correlate with the reported transcriptomic basal‐like and classical pancreatic tumor subtypes (Figure 1E, Subtypes in orange and blue). To assess whether IC3 is driven by an ISR PDA subtype, TE for ATF5, JUN and ATF4 mRNAs were plotted in five tumors with the most positive (ISR‐high) or the most negative (ISR‐low) association with IC3. This revealed a striking association between TE of these transcripts and ISR high tumors (Figure 1F), further confirmed by RT‐qPCR for ATF4 (Figure S1C). Comparing IC3 to the signatures of ISR [17] and recently described split‐ISR [18] did not allow to identify the underlying mechanism of IC3 specifically (Figure S1D,E). Altogether, this study identifies a new tumor subset characterized by a translational profile consistent with ISR activation.

### 2.2 Translation Properties of ISR‐Activated Cells

**FIGURE 2:**
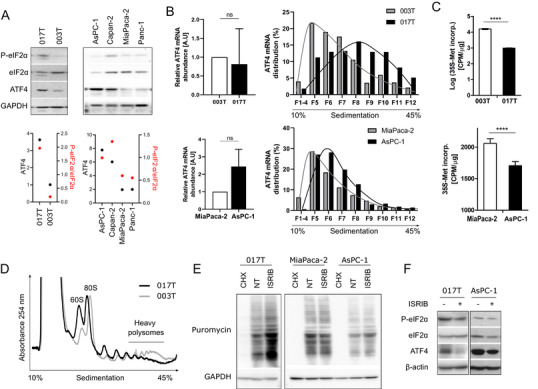
Translation properties of ISR‐activated cells (A) Western blot analysis (and quantification) of extract from PDX‐derived cell lines and from commonly‐used PDA cell lines. (B) Abundance and polysome distribution of ATF4 mRNA. (C) Protein synthesis rate ([^35^S]‐methionine incorporation). (D) Polysome profiles of PDX‐derived cells. (E) Puromycin incorporation visualized through western blot. (F) Western blot analysis of ISR‐activated cells treated with ISRIB.

Two primary PDX‐derived cancer cultures, one of each ISR‐high (017T) and ISR‐low (003T) tumors, were used to characterize the IC3 PDA phenotype. This revealed elevated levels of ATF4 protein expression and of eIF2α phosphorylation in ISR‐high 017T cells (Figure 2A). Comparing commonly used pancreatic cancer cell lines, AsPC‐1 cells displayed similar characteristics as 017T cells (Figure 2A). Hereafter, AsPC‐1 and 017T are therefore referred to as “ISR‐activated” cells, whereas MiaPaca‐2 and 003T cells are considered “translational reference” cells.

Consistent with ISR activation, ATF4 mRNA showed an enhanced TE, as evidenced by a shift toward heavy polysome fractions without change in total abundance, in ISR‐activated cells (Figure 2B), together with a reduced protein synthesis rate, as measured by [^35^S]‐methionine incorporation and heavy polysome abundance (Figure 2C,D). Conversely, distribution of olfactory receptor OR8G1 mRNA showed a relative shift to heavy polysome fractions in translation reference 003T cells (Figure S2A). To fully demonstrate the activation of ISR in 017T and AsPC‐1 cells, the ISR inhibitor ISRIB was used to stimulate ternary complex formation when eIF2α is phosphorylated [16]. As expected, ISRIB favored protein synthesis (Figure 2E) and reduced ATF4 abundance (Figure 2F) in ISR‐activated cells, but had limited effect on “translational reference” cells. Overall, these results demonstrate that the ISR‐activated phenotype is conserved from primary tumors to in vitro cell culture dishes, despite the unlimited supply of nutrients and oxygen in these culture conditions.

### 2.3 ISR‐Activated Cells are Resistant to Chemotherapies and Apoptosis

**FIGURE 3:**
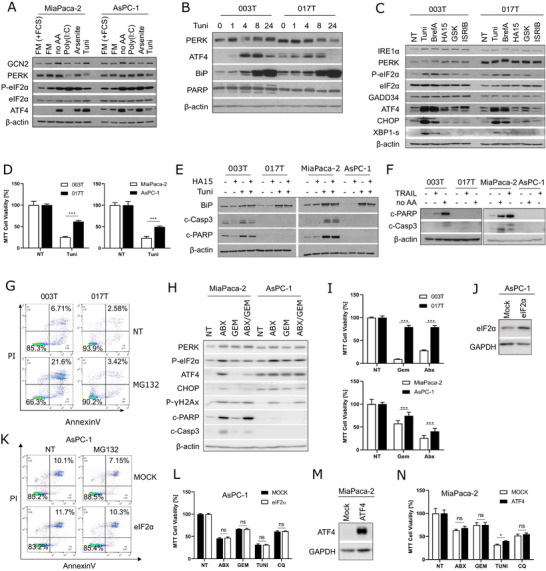
ISR‐activated cells are resistant to chemotherapies and apoptosis _(A–C) Western blot analysis. A) Cells were starved in full media without serum (FM) or without serum nor amino acids (no AA), or transfected with Poly(I:C), treated with Arsenite or Tunicamycin (Tuni). (B,C) PDX‐derived cell lines treated (B) with Tuni or (C) with Tuni, BrefeldinA (BrefA), HA15, PERK inhibitor (GSK) or ISRIB. (D) MTT assay at 48 h. E‐F) Western blot analysis of cells treated (E) with HA15, Tunicamycin or both, or (F) with TRAIL or starved (no AA). (G) Flow cytometry analysis of cells stained with Propidium Iodide (PI) and AnnexinV upon MG132 treatment. (H,I) Western blot analysis (H) and MTT assay (I) of cells treated with Abraxane (ABX), Gemcitabine (GEM), or both. (J–L) AsPC‐1 cells overexpressing eIF2α analyzed by WB (J), by flow cytometry for viability (PI) and apoptosis (Annexin V) after treatment with 250 µм MG132 for 6 h. (K) MTT assay (L) with cells treated or not with ABX, GEM, Tuni, 50 µм Chloroquine (CQ) for 48 h. Data are presented as mean ± SD (*n* = 3). (M,N) MiaPaca‐2 overexpressing ATF4, analyzed by WB (M), or by MTT assay (N) as in L.

ISR and phosphorylation of eIF2α are controlled by kinases (PERK, HRI, PKR and GCN2) and phosphatases (GADD34 and CReP) [8]. Nevertheless, none of these enzymes' expression showed strong association with the IC3 component (Figure S2B). To study if the response within each ISR arm differed between translational control and ISR‐activated cells, specific stimuli for each kinase were applied to MiaPaca‐2 and AsPC‐1 (Figure 3A). Amino acid starvation (no AA) activates GCN2. Poly(I:C) leads to PKR activation. Arsenite is thought to activate HRI, and finally, Tunicamycin (Tuni) activates PERK in the ER [19]. Strikingly, a reduced expression of ATF4 was observed in ISR‐activated AsPC‐1 cells upon Tunicamycin treatment (Figure 3A). Looking at the comparative kinetics of ISR induction, Tunicamycin was found to induce a weaker expression of ATF4, as well as PARP cleavage in ISR‐activated 017T cells (Figure 3B). Treatment with other PERK inducers, such as BrefeldinA or the BiP inhibitor HA15 [20], also hampered the induction of ATF4 in 017T cells (Figure 3C). PERK kinase was not activated under standard culture conditions, as evidenced by the lack of change in eIF2α phosphorylation following treatment with a low dose of the PERK inhibitor (GSK). However, treatment with the GCN2 inhibitor A‐92 significantly reduced eIF2α phosphorylation (Figure 3C; Figure S3A). Finally, ATF4 abundance decreased in 017T cells upon ISRIB treatment (Figure 3C). These data suggest that ATF4 expression in ISR‐activated cells may be mediated by GCN2 – eIF2α‐phosphorylation.

Consistent with the altered response to PERK inducers, ISR‐activated cells were found to be more resistant to Tunicamycin (Figure 3D; Figure S3B). Indeed, neither caspase‐3 nor PARP were cleaved in ISR‐activated cells upon treatment with HA15, Tunicamycin (Figure 3E), TRAIL, or amino acid starvation (Figure 3F). Apoptosis resistance of 017T cells was also confirmed by Annexin V labeling, upon MG132 treatment (Figure 3G). Chemotherapy combining Gemcitabine (Gem) with Abraxane (Abx) was also tested [21]. Abx induced a much stronger ISR compared to Gem in MiaPaca‐2 (Figure 3H); also Abx and Gem/Abx induced similar DNA damage (phospho‐γH2AX), yet apoptosis was only induced in MiaPaca‐2 cells (Figure 3H). Finally, chemoresistance of ISR‐activated cells was confirmed by cell viability assays (Figure 3I; Figure S3C). Taken together, these data highlight the remarkable resistance to apoptosis and to chemotherapy of ISR‐activated cells. In the face of this non‐canonical response to ISR induction, we examined the induction of various ATF4 targets in ISR‐activated and “translational reference” cells. Examining the expression of CHOP, GADD34, Beclin1, ATG13, and PCK2 did not reveal any difference between cell types, except for a global downregulation of GADD34 in ISR‐activated (Figure S3D,E).

### 2.4 Low eIF2α Abundance does not Mediate the ISR‐Activated Cell Phenotype

Although ISR‐activated cells constitutively express ATF4, results shown in the previous section point out how ISR‐related stress (such as chemotherapy) does not lead to ATF4‐CHOP mediated cell death. This suggests an impairment of ATF4 transcriptional programs, such as the expression of autophagosome components, like LC3B. Therefore, we tested the autophagy dependency of ISR‐activated cells using the autophagy inhibitor, chloroquine (CQ) and its ability to induce cell death, in combination with amino acid starvation. ISR‐activated cells were resistant to apoptosis induced by CQ and/or amino acid depletion (no AA) (Figure S3F,G). Quantification of autophagosome accumulation by LC3B immunofluorescence indicated that CQ effectively induced autophagosome formation in all tested cell lines with no obvious dependence on the ISR‐activated status (Figure S3H). Altogether, these results demonstrate that autophagy is dispensable for ISR‐activated cells.

Since ISR‐activated cells display a high basal expression of ATF4 associated with limited amounts of eIF2α (Figure 2, 3), we investigated whether overexpression of ATF4 was sufficient to induce the ISR‐activated phenotype or, conversely, if increasing the amount of eIF2α would reverse the ISR‐activated phenotype. eIF2α‐overexpressing AsPC‐1 cells demonstrated, as expected, an enhanced protein synthesis, yet did not develop altered resistance to autophagy or to apoptosis (Figure 3J–L, Figure S3I,J). Similar observations were made with ATF4‐overexpressing MiaPaca‐2 cells (Figure 3M,N; Figure S3K). The possible involvement of split‐ISR [18] in ISR‐activated cells was also ruled out because the abundance of neither the eIF2Bε nor the PCK2 proteins was correlated with ISR activation status. This suggests that only the canonical ISR is activated in these cancer cells (Figure S3D,E). Taken together, these results suggest that high ATF4 expression is a consequence rather than a cause of the phenotype observed in ISR‐activated cancer cells.

### 2.5 ISR‐Activated Cells Show Impaired Serine Metabolism

**FIGURE 4:**
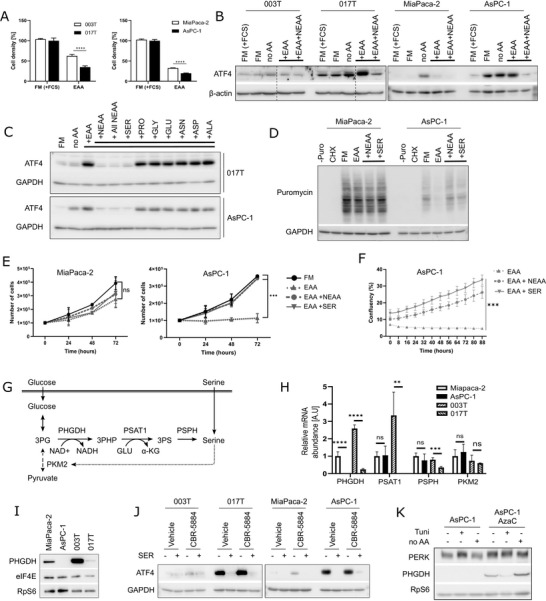
ISR‐activated cells have impaired serine biosynthesis pathway (A) Crystal Violet assay with cells in full media with serum (FM+FCS) or only with essential AA (EAA) for 48 h. (B,C) Western blot on cells grown in FM+FCS or starved in media without AA (no AA) for 2 h then B) stimulated for 4 h with EAA and with all non‐essential AA (NEAA) or C) with the indicated NEAA. (D) Puromycin incorporation in cells treated with CHX or starved in media with only EAA, supplemented with all NEAA or serine. (E,F) Proliferation assay (E) and Cell confluence (Incucyte live‐cell imaging) (F) of MiaPaca‐2 and AsPC‐1 cells cultured in the indicated medium. (G) Schematic of the serine biosynthesis pathway (SSP). (H) mRNA abundance of SSP enzymes (RT‐qPCR). (I–K) Western blot analysis of (I) cells in FM+FCS, (J) cells treated with CBR‐5884 (PHGDH inhibitor) in media with EAA, supplemented with serine, (K) AsPC‐1 cells cultured with Azacitidine (AzaC) for 3 passages, then treated with Tunicamycin (Tuni) or starved without AA.

Considering that the GCN2 inhibitor reduces eIF2α phosphorylation in ISR‐activated cells under normal growth conditions, for which autophagy appears dispensable (Figure S3A,G), we thought that exogenous nutrients could be critical for their growth. The GCN2‐eIF2α‐ATF4 pathway controls several non‐essential amino acid (NEAA) metabolic pathways, including serine, cysteine, proline, or alanine synthesis [7]. Interestingly, ISR‐activated cells showed lower growth capacity after incubation in minimal essential media (MEM) containing only essential amino acids (EAA), (Figure 4A). Upon complete amino acid deprivation in HBSS (no AA), ATF4 induction was observed in all PDA cells (Figure 4B). The sole supplementation for 4 h with essential AA (EAA) was sufficient to reduce ATF4 expression in “translational reference” cells. Conversely, both essential and non‐essential AA (EAA+NEAA) were necessary to decrease ATF4 expression in ISR‐activated cells. HBSS, supplemented with EAA and individual NEAA, allowed the identification of serine as a critical AA for ISR‐activated cells to decrease ATF4 expression (Figure 4C). Surprisingly, even though glycine and serine can interconvert through the enzymes SHMT1/2 [22], supplementation with glycine was not sufficient to reduce ATF4 in ISR‐activated cells (Figure S4A). Analysis of ATF4 expression under nutrient restriction with EAA showed an inverse correlation with protein synthesis (Figure 4C,D), which was fully recovered by the addition of NEAA (serine, proline, glutamate, glycine, asparagine, aspartate, alanine), or serine alone. These findings were confirmed by cell growth (Figure 4E) and Incucyte density analysis in similar conditions (Figure 4F).

These results suggest an impairment of the serine biosynthesis pathway (SSP) in ISR‐activated cells (Figure 4G). Indeed, phosphoglycerate dehydrogenase (PHGDH) mRNA and protein levels were reduced in ISR‐activated cells (Figure 4H,I). Furthermore, treatment with a PHGDH inhibitor (CBR‐5884) in media lacking serine (MEM) strongly induced ATF4 expression in “translational reference” cells, whereas it had no effect in ISR‐activated cells (Figure 4J).

Overall, ISR‐activated cells do not require addition of other NEAA than serine for growth and protein synthesis. Importantly, addition of formate and glycine to MEM (EAA) leads to reduction of ATF4 and eIF2α phosphorylation, similarly to addition of serine, in AsPC‐1 cells (Figure S4B,C). Furthermore, it partially restored cell growth and protein synthesis, whereas glycine addition alone had no effect (Figure S4D,E), demonstrating that fueling the folate cycle with formate could sustain serine‐dependent cell proliferation [23]. Considering the described role of ATF4 as transcriptional activator of the PHGDH promoter [24], we investigated DNA methylation of PHGDH gene in ISR‐activated cells. Indeed, demethylating agent 5‐azacytidine treatment led to consistent re‐expression of PHGDH, which was further induced upon EAA removal (Figure 4K). Altogether, these data demonstrate that ISR‐activated cells display a serine auxotrophy due to a lack of PHGDH expression. Moreover, the single serine supplementation is sufficient to rescue protein synthesis and cell growth.

### 2.6 Rewiring of the Transsulfuration Pathway Limits Serine Consumption in ISR‐Activated Cells

**FIGURE 5:**
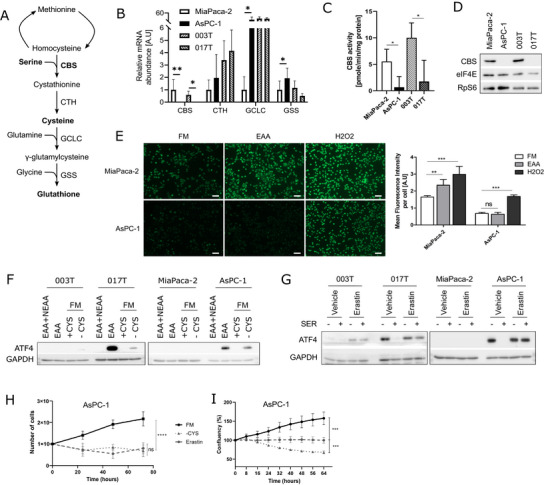
Rewiring of transsulfuration pathway limits serine consumption in ISR‐activated cells (A) Schematic of the transsulfuration pathway (TSS). (B) mRNA abundance of TSS enzymes (RT‐qPCR). (C) CBS activity. (D) Western blot analysis of four cell lines. (E) DCFDA staining of cells and Mean fluorescence intensity in full media (FM), only with essential AA (EAA) or with H2O2. (F,G) Western blot analysis of (F) cells incubated in media with only EAA or with NEAA (EAA+NEAA), in FM with or without cysteine (‐CYS), (G) cells treated with Erastin (xCT inhibitor) in media with only EAA (MEM) with or without serine. (H,I) Proliferation assay (H) and confluence analysis (I) of AsPC‐1 cells cultured in the indicated medium: full media (FM), media without cysteine (‐CYS) and FM with Erastin.

Serine is a key metabolite for the first step of the transsulfuration (TSS) pathway (Figure 5A), one essential metabolic pathway for glutathione formation and maintenance of low cellular concentrations of reactive oxygen species (ROS) [25]. Considering the serine dependency of ISR‐activated cells, expression of enzymes from the TSS pathway was examined. The Cystathionine β synthase (CBS) transcript, protein and activity were drastically down‐regulated in ISR‐activated cells (Figure 5B–D). Interestingly, and despite the absence of CBS, we found that AsPC‐1 cells had a lower basal ROS level than MiaPaca‐2 cells. Moreover, ROS level increased in MiaPaca‐2, but not in AsPC‐1, cells when cultured in MEM (EAA) (Figure 5E). In addition, culturing AsPC‐1 cells with NAC, a potent ROS scavenger, had no effect on cell growth (Figure S4F). Thus, the TSS pathway appears functional in ISR‐activated cells without serine supplementation, suggesting a need for extracellular cysteine to bypass CBS depletion (Figure 5A). In fact, after cysteine depletion in the growth medium of ISR‐activated cells or using Erastin, an inhibitor of the cysteine transporter xCT, ATF4 expression was induced in ISR‐activated cells (Figure 5F,G), conversely to cell growth (Figure 5H,I). Altogether, these data show that ISR‐activated cells present an impairment of the first step of the TSS pathway requiring serine, thereby revealing an additional dependency on cysteine.

### 2.7 Lack of PHGDH and CBS are Markers of ISR‐Activated Cells In Vitro

**FIGURE 6:**
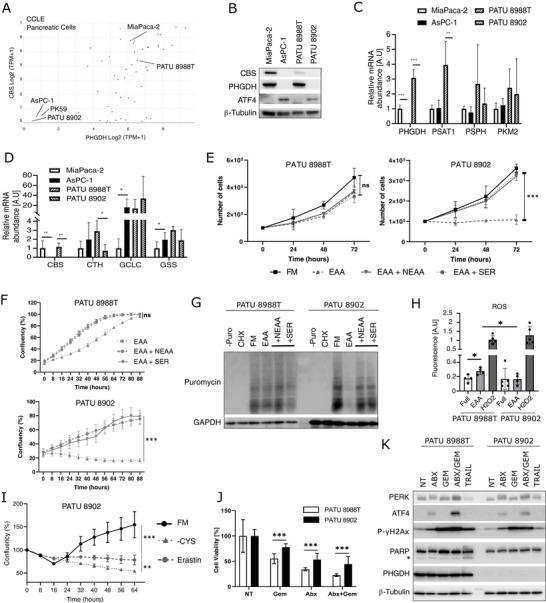
Absence of PHGDH and CBS are markers in ISR‐activated cells (A) Plot of PHGDH and CBS mRNA expression among CCLE PDA cancer cells. (B) Western blot analysis. (C,D) RT‐qPCR analysis of mRNA encoding for SSP (C) and TSS (D) enzymes. (E,F) Proliferation assay (E) and Cell confluence analysis (F) of cells cultured in full media (FM), in media with essential AA (EAA) or supplemented with non‐essential AA (NEAA) or with serine. (G) Puromycin incorporation in cells treated with CHX, starved in media with only EAA, supplemented with NEAA or serine (SER). (H) DCFDA Mean fluorescence intensity. (I) Confluence analysis of cells cultured in the full media (FM), without cysteine (‐CYS) and FM with Erastin. (J,K) Crystal violet assay (J) and Western blot analysis (K) of cells treated with Gemcitabine (Gem), Abraxane (Abx) or both. Trail induces apoptosis as visualized by PARP cleavage (*).

PHGDH and CBS downregulation is likely responsible for the double auxotrophy of ISR‐activated cells (Figures 4 and 5). Exploring the CCLE database [26] for pancreatic adenocarcinoma cells, we identified PATU 8902 and PK59 cell lines with similar expression patterns as ISR‐activated cells, and used them to corroborate whether they harbored an ISR‐activated phenotype (Figure 6A). We further characterized PATU 8902, together with the related PATU 8988T cells, which resembled MiaPaca‐2 “translational reference” cells. At first, we confirmed the lack of PHGDH and CBS in PATU 8902 cells, in association with the constitutive expression of ATF4 in full media (Figure 6B).

Further exploration of the SSP and TSS pathways by RT‐qPCR did not reveal any additionnal common variations between AsPC‐1 and PATU 8902 cells (Figure 6C,D), which corroborated CCLE RNA‐seq Data (Figure S5A,B). Similar to AsPC‐1 and 017T cells, PATU 8902 cells were fully auxotrophic for serine only, and do not require other NEAA for growth and protein synthesis (Figure 6E–G). PATU 8902 cells also showed reduced eIF2α‐phosphorylation and increased protein synthesis and cell proliferation when cultured with EAA supplemented with glycine and formate (Figure S5C–E). Moreover, ROS levels in PATU 8902 cells remained constant upon culture without NEAA, as opposed to PATU 8988T cells, which rely on serine to produce cysteine (Figure 6H). In the absence of CBS, PATU 8902 cells are unable to grow in cysteine‐depleted media or upon Erastin treatment (Figure 6I). Autophagosome abundance was similar between PATU 8988T and PATU 8902 cells (Figure S5F). In line with this metabolic dependency, patients expressing low levels of both PHGDH and CBS display an improved overall survival in two independent cohorts (Figure S5G) in accordance with previous reports on each marker [27‐–29]. Finally, treatment with Gemcitabine, Abraxane, or their combination revealed that PATU 8902 cells also exhibited resistance to chemotherapies and apoptosis (Figure 6J–K; Figure S5H). Given the relationship between the ISR‐activated phenotype, amino acid metabolism and PHGDH expression, we investigated the role of mTOR signaling. No significant differences in the phosphorylation of 4E‐BP1, S6, or S6K1 were observed between AsPC‐1, PATU 8902, and 017T ISR‐activated cells and “translational reference” cells (Figure S5I). Overall, the maintenance of most ISR‐activated features in PATU 8902 cells indicates that the absence of PHGDH and CBS can be used as markers for ISR‐activated PDA cells. This finding is consistent with the inverse correlation between the expression level of the ISR‐activated component IC3 and PHGDH mRNA (Figure S1E).

### 2.8 Specific CAFs Support ISR‐Activated Cell Growth in Serine‐Deprived Environment

**FIGURE 7:**
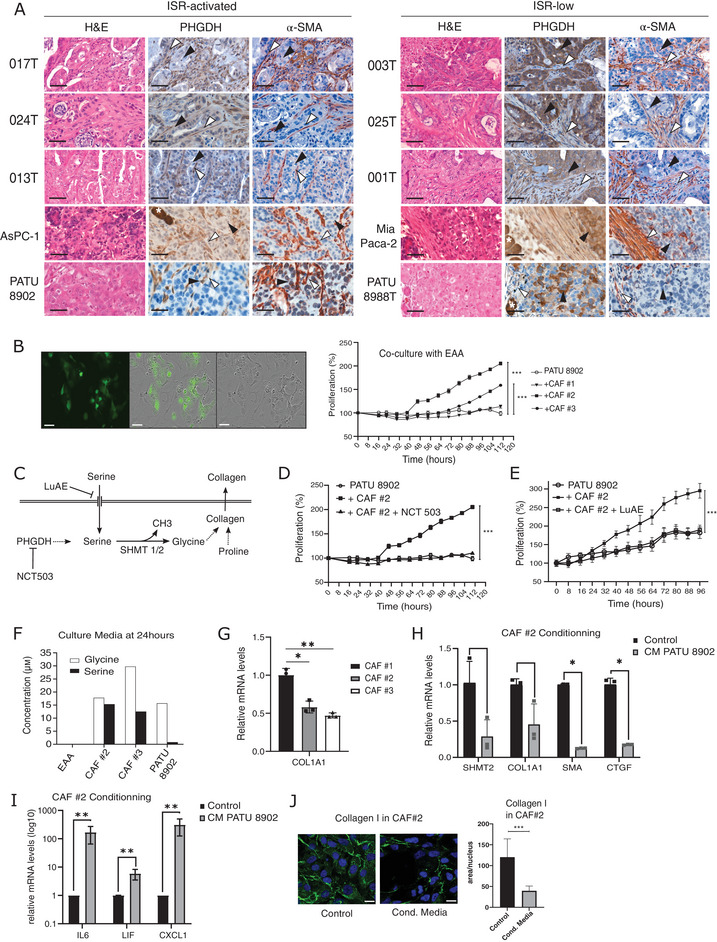
Specific CAFs sustain serine‐dependent growth of ISR‐activated cells (A) Representative hematoxylin/eosin (H&E) and immunohistochemistry (IHC) stainings of PHGDH, and αSMA on slices from ISR‐activated and ISR‐low PDX as well as Pancreatic‐grafted cells. Black arrowheads: Cancer cells, white arrowheads: αSMA‐positive cells (CAFs). White asterisk: acinar cells. (B) Representative image of co‐culture with GFP‐PATU 8902 and unlabeled CAF #2. Proliferation of GFP cells monitored by live‐cell imaging alone or in co‐culture with different CAFs. (C) Schematic of serine origin and conversion in glycine for the synthesis of collagens. (D,E) Proliferation of GFP cells in co‐culture with CAF #2 with (D) NCT 503 or (E) LuAE 00527. (F) Analysis of exogenous serine and glycine concentration produced by cells upon culture with EAA for 24 h. (G) COL1A1 mRNA abundance in CAFs (RT‐qPCR). (H,I) CAF #2 were cultured for 24 h with PATU 8902 conditioned‐media (CM PATU 8902) H‐I) Abundance of SHMT2, COL1A1, SMA, CTGF, IL6, LIF and CXCL1 mRNA. (J) Collagen expression measured by immunofluorescence.

Facing the marked auxotrophy of ISR‐activated cells, we questioned the origin of serine in the harsh tumor microenvironment of PDA. We analyzed PHGDH expression by immunohistochemistry (IHC) on the initial ISR‐activated and ISR‐low PDX samples, including 003T and 0017T, as well as on orthotopically‐grafted MiaPaca‐2, AsPC‐1, PATU 8988T and PATU 8902 cells into nude mice. A lower expression of PHGDH was detected in ISR‐activated tumors (Figure 7A), confirming the relationship between serine auxotrophy and ISR activation. Interestingly, in ISR‐activated tumors, stromal expression of PHGDH appeared to be higher than in cancer cells, especially in fibroblastic cells, visualized by αSMA staining. Similar analyses were performed on samples from a larger cohort of PDX samples [30], confirming the presence of PHGDH and αSMA double‐positive stromal cells surrounding PHGDH‐negative tumor cells (Figure S6A), suggesting that CAFs could provide serine. Importantly, PHGDH expression was not correlated to basal‐like and classical differentiation status, in neither PDX nor patient samples (Figure S6B,C). Thus, the serine‐dependent growth of GFP‐expressing PATU 8902 ISR‐activated cells was monitored by video microscopy (Incucyte) in co‐culture with CAFs originated from 3 different PDA patients. PATU 8902 cells did not grow in MEM lacking NEAA, nor in co‐culture with CAF #1 (Figure 7B). Conversely, co‐culture with CAF #2 and #3 favored growth of PATU 8902, suggesting the production of serine. Blocking serine transporters with LuAE (LU AE00527) reduced PATU 8902 cell growth when cultured in MEM with serine, conversely to PHGDH inhibitor NCT503 (Figure 7B–D; Figure S6D), consistent with the absence of PHGDH expression in these cells. When co‐cultured with CAF #2 (Figure 7D,E) or CAF #3 (Figure S6E,F), PATU 8902 cell proliferation was blocked by NCT503 or LuAE inhibitors. This indicates that PHGDH activity from CAFs, as well as serine flux are required for PATU 8902 cell growth. Subsequently, up to 15µм of serine and 30µм of glycine were detected in MEM media conditioned by CAF #2 and #3 (Figure 7F), demonstrating the capacity of CAFs to produce serine for ISR‐activated cell growth.

Comparing expression of enzymes from SSP and TSS revealed no major differences in serine biosynthetic enzyme expression between CAF #2 and CAF #3 vs. CAF #1 (Figure S6G). Hence to identify the mechanism that enables serine production, we sought to explore further whether serine could come as a byproduct of CAF functions as professional collagen I producing cells [31]. Collagen I is composed about 30% of glycine, generated from serine via SHMT1/2 enzymes (Figure 7C). SHMT2 was the dominant isoform, expressed about 100‐fold higher than SHMT1 in all CAFs (Figure S6G).

Interestingly, expression of COL1A1 mRNA was reduced in CAF #2 and #3 as opposed to CAF #1 (1.7‐ and 2.1‐fold reduction, respectively) (Figure 7G), suggesting that CAF #2 and #3 metabolism is less prone for consuming glycine for collagen synthesis. Thus, we next examined whether ISR‐activated cells could reprogram CAF metabolism to favor serine release. Using conditioned media from PATU 8902 cells, we found that SHMT2 and COL1A1 mRNA were reduced in CAF #2 (Figure 7H–I) and CAF #3 (Figure S6H–I). In parallel, increased expression of iCAF markers (IL6, LIF, and CXCL1) and reduced expression of myCAF markers (SMA and CTGF) were observed, indicative of a shift from a myCAF to an iCAF phenotype [7]. Phenotypically, this shift was associated with decreased collagen I deposition in CAF #2 (Figure 7J) and CAF #3 (Figure S6J). These findings suggest that reduced collagen synthesis lowers glycine consumption, thereby increasing the intracellular serine pool and/or lowering its conversion to glycine. Collectively, our results demonstrate that CAFs can supply serine to sustain ISR‐activated PDA cell growth. This metabolic symbiosis is actively driven by ISR‐activated cancer cells, which reprogram CAFs toward a non‐myCAF phenotype with reduced collagen production, ultimately enhancing serine availability in the tumor microenvironment.

## 3 Conclusion

Alterations of protein synthesis in cancer cells are now a well‐accepted notion. Many translation initiation factors favor cell transformation, whereas some translational regulators, including PDCD4 and 4E‐BP1, are considered as tumor suppressors [9, 32]. Exploring cancer cell mRNA translation in the context of the whole PDA tumor has been exceptionally challenging and technologically limited, until recently. Here, we performed a transcriptome‐wide analysis of translation in PDA solid tumors, to identify novel tumor subtypes with altered protein synthesis using PDX samples, considered as PDA avatars. While PDX models remain invaluable for cancer research, interspecies signaling differences can affect the fidelity of tumor–stroma dialogues. Using unbiased bioinformatics approaches, translatome analysis uncovered a subset of tumors, distinct from transcriptome‐based basal‐like and classical subtypes. These tumors are characterized by a low protein synthesis rate and a persistent activation of the ISR pathway, as evidenced by translational upregulation of mRNA encoding ATF4, ATF5 and JUN. Functional characterization of the ISR‐activated PDX‐derived cancer cells revealed a strong resistance to apoptosis and to most chemotherapies used on PDA patients. Concomitantly, ISR‐activated cells fail to activate conventional ATF4‐dependent transcriptional programs, such as apoptosis, SSP or TSS. Finally, ISR‐activated cells bear a dual amino acid auxotrophy to serine and cysteine. The defect in SSP, associated with the lack of PHGDH, coincides with a rewiring of the TSS pathway, where serine is no longer used to produce cysteine, due to the absence of CBS. These metabolic dependencies are in accordance with the improved overall survival for PDA patients expressing low PHGDH and CBS levels. Finally, serine‐producing CAFs act as important contributors to cancer‐cell survival despite their metabolic vulnerability.

Defining PDA vulnerabilities to improve patient treatment, objective response rate, and overall survival, is the holy grail of molecular profiling [21]. Here, translatome analysis identified ISR‐activated cancer cells, whose phenotype is associated with resistance to apoptosis upon treatment with Gemcitabine, *nab*‐Paclitaxel, 5‐FU and TRAIL. Importantly, ISR‐activated cells respond to most stresses with growth arrest and a marked reduction in protein synthesis. This suppression of mRNA translation, driven by elevated eIF2α phosphorylation, likely reduces ATP consumption and mitochondrial activity, as suggested in the literature [29], placing cells into a dormant‐like state, similar to persistent cancer cell populations [33]. Given their resistance profile, which obviously extends to PHGDH inhibitors [34], ISR activation status and PHGDH expression should be considered for patient stratification in clinical trials, particularly those evaluating PHGDH inhibitors or serine/glycine‐restricted diets [35].

The ISR‐activated phenotype likely arises from serine auxotrophy, as sustained ISR activation occurs following PHGDH inhibition. Critically, targeting eIF2α phosphorylation with ISRIB or eIF2α overexpression does not reverse ISR‐activated phenotype, indicating that persistent ISR is a consequence, not a cause, of serine deprivation and PHGDH loss. Although PHGDH's role in chemoresistance is complex, recent studies report that its loss correlates with chemoresistance in ovarian cancer [36]. Understanding PHGDH's role in chemoresistance may thus be critical for guiding PDA treatment strategies, particularly given its epigenetic regulation, since demethylation restores PHGDH expression, and promoter methylation is evident in the CCLE database [https://depmap.org/portal[26]]. Paradoxically, although ATF4‐driven ISR normally orchestrates adaptive responses, ISR‐activated cells show incomplete activation of ATF4‐dependent genes. This impairment contributes to dual auxotrophy, persistent eIF2α phosphorylation, and reduced protein synthesis, while simultaneously allowing cells to survive stress by bypassing CHOP‐mediated apoptosis.

Pancreatic cancer cells are extremely sensitive to extracellular nutrient availability, especially non‐essential amino acids (NEAA) [7]. Reported dependencies on alanine, proline, cysteine and asparagine highlight the importance of metabolic reprogramming of PDA cancer cells. Nevertheless, in the context of harsh hypoxic and hypoperfused microenvironment, PDA cells must face nutrient‐deprived conditions. Here, the ISR‐activated subtype shows no dependency on NEAA other than serine, which makes them adapted for survival. Due to the absence of functional SSP, glucose carbons are likely redirected toward glycolysis and the tricarboxylic acid cycle rather than serine‐dependent anabolic routes. [29] PDA cancer cells have been described as addicted to extracellular cysteine/cystine and xCT transporter to control intracellular ROS and ferroptosis [37]. Although the role of serine and CBS in controlling ROS transiently mitigates cysteine shortage, ISR‐activated cells have completely blinded this option by suppressing CBS expression. Yet, ROS levels remain low in these cells and ROS scavenging did not improve cell growth. Whether these important metabolic shifts result in cells that are also resistant to apoptosis and chemotherapy remains to be determined.

Finally, we have addressed the question of the origin of exogenous serine and uncovered the functional implications of CAFs. Within the PDA microenvironment, CAFs are the most abundant cell type and major actors implicated in tumor chemoresistance, immune escape and ECM production [7]. CAFs can also support PDA survival by providing some components, such as alanine [38] or collagen as a source of proline [39]. Despite the marked resistance of ISR‐activated cells, serine auxotrophy emerges as a weakness. Our results demonstrate that not all CAFs can support ISR‐activated cell growth and produce serine. Modification of CAF metabolic capacity, by reducing glycine usage and collagen production, likely favors the pool of serine available for export. Additionally, CAFs could release reduced glutathione, upon cysteine shortage, which could further prevent oxidative stress in ISR‐activated cells [40]. This metabolic reprogramming promotes a shift from myCAF to iCAF phenotype, which may represent a general stromal adaptation to metabolic stress [7, 41].

Understanding how ISR‐activated cells reshape their microenvironment as serine suppliers should guide future investigations. Because multiple stromal cell types may participate in this process, including neurons [29], identifying and targeting these interactions could open new avenues for treatment. Stromal reprogramming emerges as a recurrent mechanism in PDA biology and remains a promising frontier for the development of microenvironment‐based interventions [7].

## 4 Experimental Section/Methods

### 4.1 PDX Polysomes Purification

Fragments of 27 pancreatic PDX [13] were ground in liquid nitrogen using a BioPulverizer. Around 100 g of tumor powder were lysed in 350 µL hypotonic lysis buffer (5 mм Tris‐HCl pH 7.5, 2.5 mм MgCl2, 1.5 mm KCl, 100 µg/ml cycloheximide, 2 mм DTT, 0.5% Triton X‐100, 0.5% sodium deoxycholate and 1mм Ribonucleoside vanadyl complexes). 250 µL of lysates were loaded onto a 5 mL discontinuous 5%‐34%‐55% sucrose gradient (20 mм HEPES‐KOH pH 7.6, 100 mм KCl, 5 mм MgCl2) and centrifuged at 45 000 rpm (SW 55 Ti rotor, Beckman Coulter, Inc.) (adapted from [42] for 45 min at 4°C per groups of 6 including a cellular control for each batch). Fractions 8 and 9 containing mRNAs associated to more than 3 ribosomes, were pooled and purified using TRIzol‐LS (Thermo Fisher Scientific) in parallel to 50 µL of the total lysate.

### 4.2 Cell Treatment

500 000 cells were seeded in 6‐well plates and grown overnight. Cells were treated or not with indicated concentration of drugs in full media, HBSS (containing no AA, #H6648, Sigma), MEM referred as to EAA medium in the manuscript (containing no NEAA, #M2279, Sigma,) or DMEM (cysteine‐, methionine‐, glutamine‐free media #D0422, Sigma) supplemented or not with essential/non‐essential amino acid mixtures (#M5550, #M7145, Sigma), or individual amino acids. L‐alanine, L‐aspartate, L‐asparagine, L‐glutamate, glycine, L‐proline, L‐serine, L‐methionine and L‐cystine were purchased from Sigma‐Aldrich.

### 4.3 Cell Polysome Profiling and RNA Isolation

Cells were cultivated in 15 mm culture dishes. At ∼80% confluence, cells were treated with 100 µm cycloheximide (CHX) for 5 min before being harvested on ice. Cells were washed twice with cold PBS with 100 µм cycloheximide and lysed in the hypotonic lysis buffer described above. After normalizing the RNA concentration, lysates were loaded onto a 5 mL linear 10%‐45% gradient, and centrifuged at 45 000 rpm for 45 min at 4°C. The absorbance at 254 nm was continuously recorded using an ISCO fractionator and 12 fractions of ∼400 µL were collected along the fractionation. The first four fractions were pooled before mRNA isolation of all fractions and the total lysate, using TRIzol‐LS (Thermo Fisher Scientific).

### 4.4 Compounds Used

Chemical compounds used in cell culture experiments, including concentrations and treatment durations (unless otherwise stated) are listed below: ISRIB (500nм, 6 h) (#SML‐0843), Poly(I:C) (10 µg/mL, 5 h) (#I3036), Tunicamycin (8 µg/mL, 6 h) (#T7765), MG132 (250µм, 6 h) (#C2211), BrefeldinA (0.5 µg/mL, 4 h) (#B7651), GSK2606414 (50nм, 6 h) (#516535), Chloroquine (50µм) (#C6628), CBR‐5884 (30µм) (SML‐1656), NCT 503 (2.5µм) (SML1659), Arsenite (500µм, 1 h) (S#7400), H2O2 (#H1009), N‐Acetyl‐Cysteine (#A7250) and Erastin (2µм, 6 h) (#329600) were obtained from Sigma Aldrich and Trail (100 ng/mL, 16 h) (#310‐04) from Peprotech. A‐92 (1µм, 6 h) (HY‐100877) was obtained from MedChemExpress. BiP inhibitor HA15 (10µм, 4 h) was described previously [20]. Compounds were used in full media with serum, unless indicated. Nab‐paclitaxel/ABRAXANE (5µм) (Celgene), Gemcitabine (100µм) (Sandoz) and Azacitidine (1µм) (Vidaza, Celgene) were provided by the Pharmacy of “Institut Universitaire du Cancer Toulouse”. Lu AE00527 (50µм) was provided by Lundbeck (Denmark).

### 4.5 RT‐qPCR

Concentrations of RNA were measured using a NanoDrop (ND‐1000 from Thermo Fisher Scientific). 1 µg of RNA was first reverse transcribed with RevertAid H Minus Reverse Transcriptase (Invitrogen) using random hexamer primers (Invitrogen) according to the manufacturer's instruction. qPCR was carried out on a StepOne Plus (Applied Biosystems, Life Technologies) using SsoFast EvaGreen Supermix (BioRad) and primer concentration at 0.5 µм. For mRNA distribution across the linear gradient, equal volumes of RNAs were used to perform RT‐qPCR. The mRNA expression fold‐change was normalized using the delta‐delta Ct method with RPS16 or GAPDH as a control gene. Primers specific to human mRNAs were purchased from Integrated DNA Technologies (IDT) and are listed in the Table S1.

### 4.6 SUnSET Assay and [^35^S]‐Methionine Incorporation

500,000 cells were seeded in 6‐well plates and grown overnight. For SUnSET assays, cells were incubated with 1 µg/mL Puromycin (Sigma‐Aldrich) for 10 min before harvesting cells. Protein synthesis rate was visualized by Western blot, using the anti‐Puromycin (#MABE343; 1/5000) from Merck Millipore. Cycloheximide (#C7698, Sigma) was added 5 min before Puromycin and served as negative control. For [^35^S]‐methionine metabolic labeling, cells were incubated for 1 h in methionine‐ and cysteine‐free DMEM (#D0422, Sigma) with 10% dialyzed serum (#26400044, Gibco). The medium was then replaced with methionine‐ and cysteine‐free DMEM containing [^35^S]‐protein labeling mix (20 µCi/mL, EasyTag EXPRESS^35^S, Perking Elmer). After 30 min, cells were washed with cold PBS and lysed in buffer (see Preparation of Cell extracts and Western blots), and radioactivity incorporated into the TCA precipitable material was measured. Radioactivity (CPM) was normalized to protein concentration.

### 4.7 Viability Assay

Cell abundance was measured by MTT assay or Crystal violet staining. 6000 cells were plated in 96‐well plates and grown overnight. Cells were treated with indicated drugs in at least quadruplicates. 48 h later, MTT was added to each well and incubated at 37°C for 3 h. After eliminating the media, formazan crystals were dissolved in 100 µL of DMSO. For crystal violet staining, cells were fixed with 3.7% formalin then incubated in crystal violet for 20 min. After cell wash with H_2_O, cells were lysed in 10% acetic acid. The absorbance of each well was read at 570 nm using a microplate spectrophotometer Mithras (Berthold Technology).

### 4.8 Statistics

Statistical significance was determined using two‐way ANOVA or paired/unpaired t‐test *p*‐value depending on the experiments. Correlation analysis were performed using Spearman's correlation coefficient. All values are mean ± SD. A *p*‐value <0.05 was considered statistically significant (^*^ *P*<0.05; ^**^ *P*<0.005; ^***^ *P*<0.0005; ^****^ *P*<0.00001), unless indicated otherwise.

## Author Contributions

S.S., M.L., M.L.B, Ch.J., L.F., I.B, B.F. and Y.M. designed and carried experiments. R.N. analyzed RNAseq data and performed computational biology and independent component analysis. R.N., J.S., M.D. and A.B. performed computational biology analysis. Ch.J., I.B and C.N. performed orthotopic xenograft. B.F., Ma.L. and L.R. analyzed serine and glycine production and provided guidelines for serine metabolism analysis. Ca.J. provided guidelines for autophagy experiment design, LC3B IF protocol and quantification of autophagic flux. S.R. provided HA15 compound [20]. V.P provided expertise in bioinformatic analysis. A.P. and M.M. provided primary cultures of cancer‐associated fibroblasts. N.F, J.I and N.D provided PDX fragments and slices, PDX‐derived cells and associated expertise. O.L. provided expertise on polysome‐profiling in small tissue samples, translatome analysis and use of Anota2seq. S.S., S.P and Y.M. conceived the study. S.P., C.B. and Y.M. supervised the study and provided funding. S.S and Y.M. drafted the manuscript. R.N., M.L., M.L.B, Ch.J., A.B., J.S., O.L., S.P., C.B., edited the manuscript.

## Conflicts of Interest

The authors declare no conflicts of interest.

## Supporting information




**Supporting File**: advs73963‐sup‐0001‐SuppMat.pdf.

## Data Availability

The data that support the findings of this study are available from the corresponding author upon reasonable request.
