# Supplementary material for: Decoding the Integrated Stress Response of Pancreatic Cancer: Identifying a Serine‐dependent Tumor Subset Under Metabolic Relationships With CAFs
Source: Adv Sci (Weinh). 2026 Feb 17;13(18):e15740. doi: 10.1002/advs.202515740 (PMC13042921; doi:10.1002/advs.202515740)
Supplement: Supplementary file 1 — Supporting File: advs73963‐sup‐0001‐SuppMat.pdf. [file ADVS-13-e15740-s001.pdf]

Supporting Information

Decoding the integrated stress response of pancreatic cancer: identifying a serine-dependent subtype under metabolic relationships with CAFs.

*Sauyeun Shin, Mehdi Liauzun, Jacobo Solorzano, Morgane Le Bras, Christine Jean, Benjamin Fourneaux, Margaux Dore, Léa Février, Ismahane Belhabib, Alexia Brunel, Cindy Neuzillet, Marion Larroque, Carine Joffre, Stephane Rocchi, Nicolas Fraunhoffer, Aurelie Perraud, Muriel Mathonnet, Vera Pancaldi, Laetitia Linares, Juan Iovanna, Nelson Dusetti, Ola Larsson, Remy Nicolle, Stephane Pyronnet, Corinne Bousquet, Yvan Martineau**

Content:

Supplemental Materials and Methods

Supplemental Table S1

Supplemental References

Supplemental Figures

1. Supplementary Materials and Methods

Cell culture

AsPC-1 (CRL-1682) cells were cultured in RPMI-1640 medium (#R0883, Sigma-Aldrich), while MiaPaCa-2 (CRL-1420) cells were maintained in high-glucose DMEM (#D6429, Sigma-Aldrich); both cell lines were obtained from the American Type Culture Collection (ATCC). PATU 8902 (DSMZ no.: ACC 179) and PATU 8988T (DSMZ no.: ACC 162) were obtained from Dieter Saur laboratory (DKTK, Germany) and cultured in high-glucose DMEM. CAF lines (CAF#1, 2, 3) were obtained through the outgrowth method and culture in DMEM/F12 medium (# D8437- Sigma) as previously described.[1] To ensure experimental robustness, all cell lines were regularly thawed, and experiments were performed at low passage numbers to minimize phenotypic drift and avoid cross-contamination. All cell lines and CAFs were confirmed mycoplasma-free, with monthly testing performed using a detection kit (ThermoFisher). Media were supplemented with 10% (v/v) fetal calf serum (ThermoFisher Scientific), 2mM L-Glutamine (GLN) and 1% (v/v) penicillin/streptomycin (Sigma-Aldrich) at 37°C and 5% CO2. Cells derived from pancreatic PDX (003T and 017T) were grown in Serum Free Ductal Media (SFDM) as described.[2,3] GFP-expressing Patu8902 cells were obtained upon transduction with the Lv-eGFP lentiviral vector and FACS sorting to obtain a homogeneous GFP-positive population.

Proliferation assay

~100,000 cells were seeded in 12 well-plates in complete media. Once cells adhered to the plate, complete media was gently removed and cells were washed once with PBS to eliminate the serum. Cells were cultivated in the indicated medium in triplicates. After 24, 48 and 72 hours, cells were counted in 0.2% Trypan Blue solution (Sigma)

using Countness II Automated Cell Counter (Applied Biosystems, Life Technologies). Only viable cells were taken in account.

RNA sequencing

Libraries were prepared with NEBNext® Ultra™ II Directional RNA Library Prep Kit for Illumina according to the supplier recommendations (NEB). Briefly, the key stages of this protocol were successively, the removal of ribosomal RNA fraction from 80 ng of total RNA using the RiboCop rRNA Depletion Kit V1.2 (Human/Mouse/Rat) from Lexogen, a fragmentation using divalent cations under elevated temperature to obtain approximately 300bp fragments, double strand cDNA synthesis, using reverse transcriptase and random primers, and finally Illumina adapters ligation and cDNA library amplification by PCR for sequencing. Sequencing was then carried out in paired-end 75b mode with an Ilumina HiSeq 4000. Base calling was performed using the Real-Time Analysis software sequence pipeline (version 2.7.7) from Illumina with default parameters.

Translatome and Independent Component Analysis

Gene counts were normalized using TMM. The residuals of the linear model between the polysome-RNA-counts and total-RNA-counts (corresponding to Translation efficiency) were computed and subsequent analyses were only performed on the residuals of a subset of genes for which relevant linear models were obtained. This was done by selecting models with: constant error variance (using the Score Test with a minimum p-value of 1%), normally distributed residuals (using Shapiro-Wilk's test with a minimum p-value of 1%) and the absence of outliers (using Bonferroni outlier test with alpha=5%). ICA was performed on the 50% most variable (using inter quartile range) selected genes residuals using the JADE algorithm. ISR signatures were

retrieved from Andreev et al., eLife, [4] and Split-ISR signatures from Chen et al., Nature.[5] Correlation analysis was performed to assess the association between IC3 and these signatures in the residual fractions of polysome profiling RNA sequencing or total RNA, using the PDX weight in the ICA.

Preparation of Cell Extract and Western blot

Cells were washed twice in cold PBS on ice and lysed in 50 mM Tris-HCl pH 7.5, 150 mM NaCl, 2 mM EDTA, 1% NP40, 0.1% SDS and 0.5% sodium deoxycholate supplemented with EDTA-free protease inhibitor and PhosSTOP phosphatase inhibitor (Roche Applied Science), then centrifuged at 13,000 rpm for 5 minutes. Protein concentrations were measured using Protein assay reagent (Biorad) and equal amount of proteins were loaded into a 6%-15% SDS-polyacrylamide gradient gel and transferred onto nitrocellulose membrane (BioTraceNT; Pall Corp.). Membranes were washed in Tris Buffer Saline with 0.1% Tween 20 (TBS-T) and saturated in TBS-T with 5% non-fat dry milk for 20 minutes and incubated overnight in the primary antibody solution (TBS-T with 5% BSA). The following antibodies were used to detect proteins of interest: anti-ATF4 (#11815), anti-eIF2α (#2103), anti-phospho-eIF2α (#3398), anti-CHOP (#2895), anti-cleaved-caspase-3 (#9579S), anti-PARP (#9542S), anti-BiP (#3177S), anti-PERK (#3192S), anti-eIF4E (#2067S), anti-LC3B (#3868), anti-IRE1 (#3294), anti-γH2AX (#9718), anti-XBP1s (#12782), anti-PCK2 (#6924), anti-Beclin1 (#3738), anti-GCN2 (#65981), anti-pGCN2 (#94668) and anti-ATG13 (#13273) were purchased from Cell Signaling Technology, anti-GAPDH (sc-25778), anti-RpS6 (sc-74459), anti-eIF2Bε (sc-55558) and anti-GADD34 (sc-373815) were purchased from Santa Cruz Biotechnology, and anti-β-actin (clone AC-74), anti-PHGDH (HPA021241) were purchased from Sigma, and anti-CBS (ab140600) was purchased from Abcam. Mouse (#31430) and rabbit (#31460) secondary antibodies from Pierce were used at

1:10000. Signals were revealed by chemoluminescence using ECL (RevelBlot, Ozyme) and captured using Chemidoc imager (Bio-Rad). When indicated, ATF4, eIF2α and phospho-eIF2α were quantified using ImageJ (NIH).

Flow cytometry for apoptosis detection

Cells were plated in 6-well culture plates and were grown overnight. Cells were treated with 250 µM MG132 for 6 hours. Cells were washed twice with cold PBS, and then resuspended in 1x AnnexinV Binding buffer (#556454, BDbioscience) at a concentration of 1.10e6 cells/mL. 5 µL of FITC AnnexinV (#556420, BDbioscience) and 10 µL of 50 µg/mL Propidium Iodide (#556463, BDbioscience) were added to 100 µL of each cell suspension, and were incubated for 15 minutes at RT. 400 µL of 1x AnnexinV Binding buffer was added to each tube and cells were analyzed on a MACSQuant VYB (Miltenyi Biotec). A minimum of 10 000 cells were analyzed per condition. Data analysis were carried out using FlowJo v10.

CBS enzymatic activity

Cells were seeded on a 150 mm diameter tissue culture plate. At ~80% confluency, cells were washed twice with cold PBS and were collected in tubes, centrifuged at 2000 rpm for 10 minutes. Supernatents were eliminated and cell pellets were flashed frozen and stored at -80°C. CBS activity in cells were measured using the CBS activity fluorometric assay kit from BioVision (#K998) following the manufacturer’s instruction. Fluorescence (Ex/Em 368/460 nm) was measured using Clariostar (BMG Labtech) during 30 minutes.

DCFDA staining

Cells were seeded on a 6-well plate. At ~80% confluency, cells were put in the indicated media for 2 hours and then incubated with 10 µM DCFDA dye at 37°C without CO2 for 1 hr. Cells were visualized on a videomicroscope Cell Observer using the ZEN software. Images were captured arbitrarily from three different spots using the 10x /10.3 objective, and the Mean Fluorescence Intensity were quantified through ZEN and normalized on the number of cells quantified using Image J (NIH).

Orthotopic xenograft

10^6 MiaPaca-2, AsPC-1, PATU 8902 and 8988T cancer cells contained into 20 µl of PBS were injected orthotopically into the pancreas of anesthetized (Isoflurane, Isovet from Piramal Health) 8-week-old female NMRI nude mice (Charles River, France). Tumour growth was monitored by ultrasound and mice were euthanized when the tumour size reached a volume threshold of 500 mm3, according to local ethical recommendation as described.[1] Primary pancreatic tumour was removed and paraffin-embedded before being sliced and stained. All experiments were in accordance with institutional guidelines and European animal protection law and approved by the responsible government agency (Facility agreement number A31555010; Project number: AP AFIS#2I117-2019061900061441

Lentiviral expression of ATF4 and eIF2α

Mouse ATF4 cDNA was PCR-amplified and cloned into empty vector pLenti-CMV-Ires turbo-GFP-Blast using BamHI/NheI. A similar strategy was applied to human eIF2alpha cDNA using pCDNA eIF2alpha as matrix (Addgene 21807). ATF4-, eIF2α-expressing and empty lentivirus were produced and titered at the vectorology platform of the CRCT. Cells were infected at low MOI (0.5 to 2) and selected for 3 serial passages with 2µg/ml of Blasticidin.

Immunofluorescence and microscopy of LC3b

Cells were grown on 15 mm x 15 mm coverslip and treated with 50 µM Chloroquine for 4 hours. After washing twice with cold PBS on ice, cells were fixed with 3.7% formaldehyde for 10 minutes at room temperature (RT), then permeabilized and saturated in PBS supplemented with 3% BSA and 0.01% saponin during 30 minutes at RT. Cells were incubated in anti-LC3 antibody (#PM036 from MBL International Corp.) at dilution 1:800 for 45 minutes, then in secondary antibody conjugated with Alexa Fluor 488 (Invitrogen) at 1:1000 for 30 minutes after cell wash. Cells were incubated in 100 ng/mL DAPI for 10 minutes before mounting slides using the Fluorescent mounting medium (DAKO). Cells were visualized on Zeiss LSM780 confocal microscope using the ZEN software. Images were captured arbitrarily from four different spots using the 63x /1.4 objective, and the %Area of LC3 per cells were quantified using ImageJ (NIH). A color threshold was applied to quantify the total areas of green dots, and were normalized on the number of cells in the field. The mean was calculated for each condition.

Survival Analysis on Public cohorts

Impact on patient overall survival of the two proposed markers was estimated using Kaplan–Meier technique and compared with the log-rank test on Puleo[6] and ICGC[7] cohorts. For the definition of High and low PHGDHCBS groups the average of PHGDH and CBS normalized intensities was calculated, and the top and bottom 33% quantile was utilized. Overall survival was obtained from the respective publications by contacting the authors. R statistical suite (R Core Development Team) survival and survminer packages were used for calculation and visualization.

Exometabolomic analysis

CAF#2, CAF#3 and PATU8902 cells were grown in 6cm dishes. At 80% confluency, cells were washed once with 3 ml of PBS and incubated in MEM supplemented with glutaminefor 24 hrs. Conditioned media were collected and centrifuged at 2,000xg for 10min at 4°C then 10,000xg for 5 min. Supernatants were deproteinized with an addition of 200μL 30% sulfosalicylic acid. The resulting solution was vortex-mixed for 5s, incubated at room temperature for 10 min and centrifuged at 2,500rpm for 5min at RT. Supernatants were collected and stored at -80°C. A 25μL aliquot of the supernatants was then injected into the LC-MS/MS system for serine and glycine measurement. Instrumentation and conditions LC/MSMS analysis are detailed in supplementary materials.

Immunofluorescence and microscopy

Cells were grown on 15 mm x 15 mm coverslip and cultured in PATU 8902 conditioned media for 24h. After washing twice with cold PBS on ice, cells were fixed with 3.7% formaldehyde for 10 minutes at room temperature (RT), then permeabilized and saturated in PBS supplemented with 3% BSA and 0.01% saponin during 30 minutes at RT. Cells were incubated in anti-Collagen I antibody (PAI-26204 Thermo) at dilution 1:200 for 45 minutes, then in secondary antibody conjugated with Alexa Fluor 488 (Invitrogen) at 1:1000 for 30 minutes after cell wash. Cells were incubated in 100 ng/mL DAPI for 10 minutes before mounting slides using the fluorescent mounting medium (DAKO). Cells were visualized on Zeiss LSM780 confocal microscope using the ZEN software. Images were captured arbitrarily from four different spots using the 63x /1.4 objective, and the %Area of Collagen 1 per cells were quantified using ImageJ (NIH). A color threshold was applied to quantify the total areas of collagen signal and were

normalized on the number of cells in the field. The mean was calculated for each condition.

Video microscopy analysis Incucyte

A 96-well plate was seeded with 5,000 GFP-expressing PATU 8902 cancer cells per well with or without CAF lines (CAF#1, 2, 3) at a ratio of 2:1 (10,000 cells) in culture medium containing 50% DMEM, 50% DMEM/F12 supplemented with 10% FCS. 12 hours after seeding, cells were serum deprived. Cells were washed twice with PBS and placed in MEM medium supplemented with glutamine with or without the indicated amino acids at 100µM or inhibitor (NCT 503 2.5µM, LuAE 00527 50µM). Bright field images and GFP signals were captured with an Incucyte Sartorius videomicroscope, every 8 hours with 4 images per well. The Incucyte analysis software was then used to apply several masks to the acquisitions (confluency, green objects, etc.) to monitor tumor cell proliferation. For co-culture experiments, cell proliferation was measured by counting green objects per image and represented as percentage of the initial time point. Viable cell analysis was performed by seeding 5,000 cancer cells (AsPC-1, MiaPaCa-2, Patu8988T, Patu8902, 003T, 017T) per well in 96-well plates. After 12 h, cells were serum-starved and treated for 48 h. Dead cells were labeled with propidium iodide (1:1000) and imaged using an Incucyte Sartorius videomicroscope. Following 5 min incubation with 0.05% Triton X-100, all cells were imaged again. The Incucyte software quantified fluorescent objects, and cell viability was expressed as the percentage of live cells. Data represent mean ± SEM from six wells (n = 2).

Histology and immunohistochemistry

Histopathological features were determined using hematoxylin/eosin staining (H&E). Immunostaining was conducted using standard methods on formalin-fixed, paraffin-

embedded tissues. Heat antigen retrieval was performed in 10mM Citrate pH6 at 120°C for 12 min in autoclave. Antibody dilution was as follows PHGDH (HPA02124; 1/250), αSMA (ab7817;1/400). PDX samples were previously described.[2] Representative slides were imaged using 250 Flash II scanner (Pannoramic).

2. Table S1

Primer sequences for qPCR

Target	Forward sequence - 5' to 3'	Reverse Sequence - 5' to 3'
**Atf4**	ATGGGTTCTCCAGCGACAAG	GAAGGCATCCTCCTTGCTGT
**Atf5**	AAGAGAAATGAACTGGTGCAGG	AGAGGCGGCGACACTCTT
**c-Jun**	CTGCAAAGATGGAAACGACC	CAGCTTGAGCAGCCCGACGTC
**Phgdh**	TCCAAGGCACTACGCCTGTA	CTCTGCCAGGAGGCCAATCA
**Psat1**	TGGCATTAGTGTTCTTGAAATGAGT	AGCAATTCCCGCACAAGAT
**Psph**	TGCAGGTTTTGATGAGACGCA	AATCCAATGAAAGCATCAGCAGG
**Pkm2**	TGCCATGAATGTTGGCAAGGC	CCATCCGGTCAGCACAATGA
**Cbs**	GACCTCACGGAGAAGAAGCC	CATTCCCAGGATTACCCCCG
**Cth**	CAAGCTTTGAAGGCAGCACA	GCAAAGGCTCATTGTTGGTCC
**Gclc**	CACCCTCGCTTCAGTACCTT	CCGGCTTAGAAGCCCTTGAA
**Gss**	TCGAAATCAACACCATCTCTGCC	AATCAGTAGCACCAGAGCATTGGG
**Shmt1**	GGAACAGACGTTTACGGCCA	GTCTGCCATTGCACTGGTTC
**Shmt2**	ACCCCGGTACTACACCGATA	CAGACCAGCTGACCACATCT
**Col1a1**	GTGCTAAAGGTGCCAATGGT	ACCAGGTTCACCGCTGTTAC
**Slc1a4**	TGTGGACTGGATTGTGGACCG	AGTTCCTGCTCGCCTTTCTT
**Acta2**	ATGACTCAAATTATGTTTGAGACTTTCA	GTCCAGAGGCATAGAGAGACA
**Ctgf**	CTGCCCTCGCGGCTTAC	CATCCCACAGGTCTTGGAACAG
**IL6**	TCAATGAGGAGACTTGCCTG	GCTTGTTCCTCACTACTCTCAA
**Cxcl1**	CGAAGTCATAGCCACACTCAA	GATTTGTCACTGTTCAGCCACACTCAA
**Lif**	ATACGCCACCCATGCCACAA	CCCTGGCCTGTGTAATAGAGAA
**Gapdh**	GAGAGAAACCCGGGAGGCTA	ACGACCAAATCCGTTGACTC
**Rrps16**	AATGGGCTCATCAAGGTGAACGGA	TATCCACACCAGCAAATCGCTCCT

3. Supplementary References

[1] S. Zaghdoudi, E. Decaup, I. Belhabib, R. Samain, S. Cassant-Sourdy, J. Rochotte, A. Brunel, D. Schlaepfer, J. Cros, C. Neuzillet, M. Strehaiano, A. Alard, R. Tomasini, V. Rajeeve, A. Perraud, M. Mathonnet, O. M. Pearce, Y. Martineau, S. Pyronnet, C. Bousquet, C. Jean, *EMBO Molecular Medicine* **2020**, *12*, e12010.

[2] R. Nicolle, Y. Blum, L. Marisa, C. Loncle, O. Gayet, V. Moutardier, O. Turrini, M. Giovannini, B. Bian, M. Bigonnet, M. Rubis, N. Elarouci, L. Armenoult, M. Ayadi, P. Duconseil, M. Gasmi, M. Ouaissi, A. Maignan, G. Lomberk, J.-M. Boher, J. Ewald, E. Bories, J. Garnier, A. Goncalves, F. Poizat, J.-L. Raoul, V. Secq, S. Garcia, P. Grandval, M. Barraud-Blanc, E. Norguet, M. Gilabert, J.-R. Delpero, J. Roques, E. Calvo, F. Guillaumond, S. Vasseur, R. Urrutia, A. de Reyniès, N. Dusetti, J. Iovanna, *Cell Reports* **2017**, *21*, 2458.

[3] R. Nicolle, O. Gayet, P. Duconseil, C. Vanbrugghe, J. Roques, M. Bigonnet, Y. Blum, N. Elarouci, L. Armenoult, M. Ayadi, A. de Reyniès, F. Puleo, J. Augustin, J. F. Emile, M. Svrcek, T. Arsenijevic, P. Hammel, M. Giovannini, P. Grandval, L. Dahan, V. Moutardier, M. Gilabert, J. L. V. Laethem, J. B. Bachet, J. Cros, J. Iovanna, N. J. Dusetti, *Annals of Oncology* **2021**, *32*, 250.

[4] D. E. Andreev, P. B. O'Connor, C. Fahey, E. M. Kenny, I. M. Terenin, S. E. Dmitriev, P. Cormican, D. W. Morris, I. N. Shatsky, P. V. Baranov, *eLife* **2015**, *4*, e03971.

[5] C.-W. Chen, D. Papadopoli, K. J. Szkop, B.-J. Guan, M. Alzahrani, J. Wu, R. Jobava, M. M. Asraf, D. Krokowski, A. Vourekas, W. C. Merrick, A. A. Komar, A. E. Koromilas, M. Gorospe, M. J. Payea, F. Wang, B. L. L. Clayton, P. J. Tesar, A. Schaffer, A. Miron, I. Bederman, E. Jankowsky, C. Vogel, L. S. Valášek, J. D. Dinman, Y. Zhang, B. Tirosh, O. Larsson, I. Topisirovic, M. Hatzoglou, *Nature* **2025**, *641*, 1319.

[6] F. Puleo, R. Nicolle, Y. Blum, J. Cros, L. Marisa, P. Demetter, E. Quertinmont, M. Svrcek, N. Elarouci, J. Iovanna, D. Franchimont, L. Verset, M. G. Galdon, J. Devière, A. de Reyniès, P. Laurent-Puig, J.-L. Van Laethem, J.-B. Bachet, R. Maréchal, *Gastroenterology* **2018**, *155*, 1999.

[7] P. Bailey, D. K. Chang, K. Nones, A. L. Johns, A.-M. Patch, M.-C. Gingras, D. K. Miller, A. N. Christ, T. J. C. Bruxner, M. C. Quinn, C. Nourse, L. C. Murtaugh, I. Harliwong, S. Idrisoglu, S. Manning, E. Nourbakhsh, S. Wani, L. Fink, O. Holmes, V. Chin, M. J. Anderson, S. Kazakoff, C. Leonard, F. Newell, N. Waddell, S. Wood, Q. Xu, P. J. Wilson, N. Cloonan, K. S. Kassahn, D. Taylor, K. Quek, A. Robertson, L. Pantano, L. Mincarelli, L. N. Sanchez, L. Evers, J. Wu, M. Pinese, M. J. Cowley, M. D. Jones, E. K. Colvin, A. M. Nagrial, E. S. Humphrey, L. A. Chantrill, A. Mawson, J. Humphris, A. Chou, M. Pajic, C. J. Scarlett, A. V. Pinho, M. Giry-Laterriere, I. Rooman, J. S. Samra, J. G. Kench, J. A. Lovell, N. D. Merrett, C. W. Toon, K. Epari, N. Q. Nguyen, A. Barbour, N. Zeps, K. Moran-Jones, N. B. Jamieson, J. S. Graham, F. Duthie, K. Oien, J. Hair, R. Grützmann, A. Maitra, C. A. Iacobuzio-Donahue, C. L. Wolfgang, R. A. Morgan, R. T. Lawlor, V. Corbo, C. Bassi, B. Rusev, P. Capelli, R. Salvia, G. Tortora, D. Mukhopadhyay, G. M. Petersen, Australian Pancreatic Cancer Genome Initiative, D. M. Munzy, W. E. Fisher, S. A. Karim, J. R. Eshleman, R. H. Hruban, C. Pilarsky, J. P. Morton, O. J. Sansom, A. Scarpa, E. A. Musgrove, U.-M. H. Bailey, O. Hofmann, R. L. Sutherland, D. A. Wheeler, A. J. Gill, R. A. Gibbs, J. V. Pearson, N. Waddell, A. V. Biankin, S. M. Grimmond, *Nature* **2016**, *advance online publication*.

[8] N. A. Fraunhoffer, A. M. Abuelafia, M. Bigonnet, O. Gayet, J. Roques, R. Nicolle, G. Lomberk, R. Urrutia, N. Dusetti, J. Iovanna, *NPJ Precis Oncol* **2022**, *6*, 57.

Figure S1

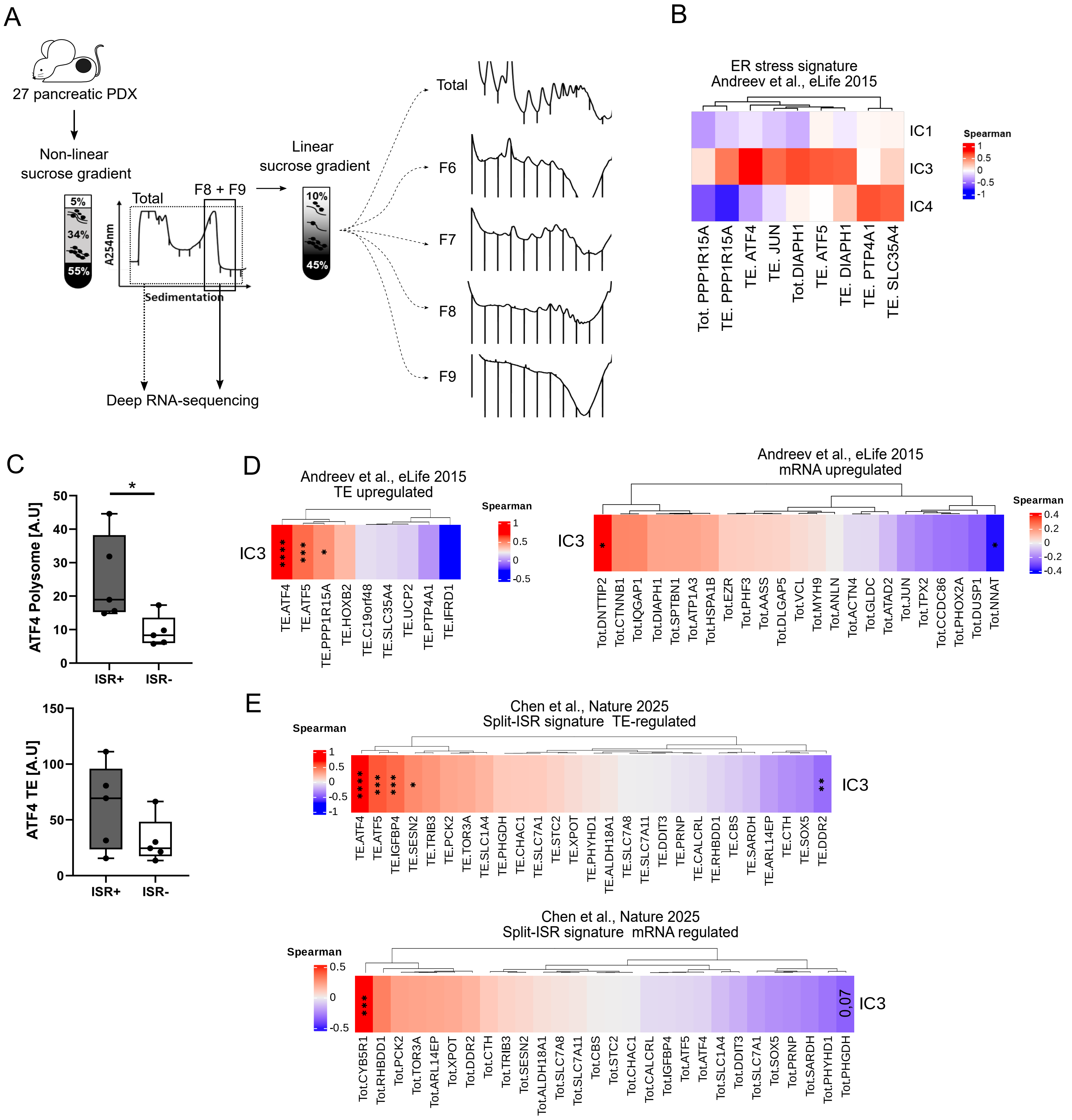
A
27 pancreatic PDX
Non-linear sucrose gradient
5%
34%
55%
Total
F8 + F9
A254nm
Sedimentation
Deep RNA-sequencing
Linear sucrose gradient
10%
45%
Total
F6
F7
F8
F9
B
ER stress signature
Andreev et al., eLife 2015
IC1
IC3
IC4
Spearman
Tot. PPP1R15A
TE. PPP1R15A
TE. ATF4
TE. JUN
Tot.DIAPH1
TE. ATF5
TE. DIAPH1
TE. PTP4A1
TE. SLC35A4
C
ATF4 Polysome [A.U]
ISR+
ISR-
ATF4 TE [A.U]
D
Andreev et al., eLife 2015
TE upregulated
IC3
Spearman
TE.ATF4
TE.ATF5
TE.PPP1R15A
TE.HOXB2
TE.C19orf48
TE.SLC35A4
TE.UCP2
TE.PTP4A1
TE.IFRD1
Andreev et al., eLife 2015
mRNA upregulated
Tot.DNTTIP2
Tot.CTNNB1
Tot.IQGAP1
Tot.DIAPH1
Tot.SPTBN1
Tot.ATP1A3
Tot.HSPA1B
Tot.EZR
Tot.PHF3
Tot.AASS
Tot.DLGAP5
Tot.VCL
Tot.MYH9
Tot.ANLN
Tot.ACTN4
Tot.GLDC
Tot.ATAD2
Tot.JUN
Tot.TPX2
Tot.CCDC86
Tot.PHOX2A
Tot.DUSP1
Tot.NNAT
E
Chen et al., Nature 2025
Split-ISR signature TE-regulated
TE.ATF4
TE.ATF5
TE.IGFBP4
TE.SESN2
TE.TRIB3
TE.PCK2
TE.TOR3A
TE.SLC1A4
TE.PHGDH
TE.CHAC1
TE.SLC7A1
TE.STC2
TE.XPOT
TE.PHYHD1
TE.ALDH18A1
TE.SLC7A8
TE.SLC7A11
TE.DDIT3
TE.PRNP
TE.CALCRL
TE.RHBDD1
TE.CBS
TE.SARDH
TE.ARL14EP
TE.CTH
TE.SOX5
TE.DDR2
Chen et al., Nature 2025
Split-ISR signature mRNA regulated
0,07
Tot.CYB5R1
Tot.RHBDD1
Tot.PCK2
Tot.TOR3A
Tot.ARL14EP
Tot.XPOT
Tot.DDR2
Tot.CTH
Tot.TRIB3
Tot.SESN2
Tot.ALDH18A1
Tot.SLC7A8
Tot.SLC7A11
Tot.CBS
Tot.STC2
Tot.CHAC1
Tot.CALCRL
Tot.IGFBP4
Tot.ATF5
Tot.ATF4
Tot.SLC1A4
Tot.DDIT3
Tot.SLC7A1
Tot.SOX5
Tot.PRNP
Tot.SARDH
Tot.PHYHD1
Tot.PHGDH

Figure S1

A) Schematic of the translatome-based PDA classification procedure. PDX lysates were loaded onto a non-linear sucrose gradient. Fraction 6-9 were collected and loaded onto a linear sucrose gradient for validation. Fractions 8-9 contain RNA associated with >3 ribosomes. B) Component association to ER stress signature. C) ATF4 mRNA abundance in the polysome fraction (F8+F9, up) and the Translation Efficiency (ratio [F8+F9]/[total extract], down) of “ISR low” (003T, 025T, 001T, 028T, 016T) and “ISR high” (024T, 009T, 013T, 017T, 020T) group. P-values were calculated using unpaired t-test (*P< 0.05). D) IC3 correlation with the mRNAs from Andreev et al. (eLife, 2015) that are upregulated at the TE level (left) or abundance level (right). E) IC3 correlation with the split-ISR specific mRNAs from Chen et al. (Nature, 2025) that are upregulated at the TE level (up) or abundance level (bottom).

(*P < 0.05, **P < 0.01, ***P < 0.001, ****P < 0.0001)

Figure S2

A

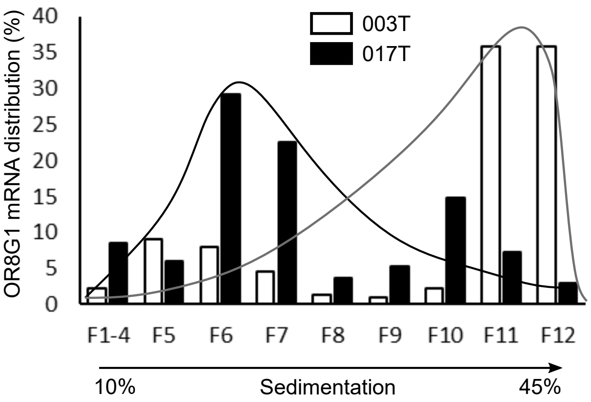

B

Transcriptional correlation with IC3

	Pearson	Spearman
GADD34	-0,33 (p=0,09)	-0,32 (p=0,100)
CREP	0,27 (p=0.17)	0,39 **(p=0.046)**
PERK	0,03 (p=0.90)	0,15 (p=0.44)
GCN2	-0,009 (p=0.97)	0,19 (p=0.34)
HRI	0,039 (p=0.85)	0,15 (p=0,46)
PKR	-0,0004 (p=0.99)	0,12 (p=0.56)

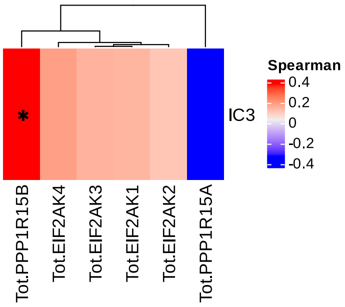

Figure S2

A) OR8G1 mRNA distribution along 10%-45% linear polysome gradient was analyzed through RT-qPCR. B) Pearson and Spearman correlations between IC3 component and total mRNA abundance (transcriptional) of eIF2α kinases PERK (EI2FAK3), GCN2 (EI2FAK4), HRI (EI2FAK1), PKR (EI2FAK2) and phosphatases GADD34 (PPP1R15A), CReP (PPP1R15B) (*$P < 0.05$). P-values >0.05 were considered statistically insignificant.

Figure S3

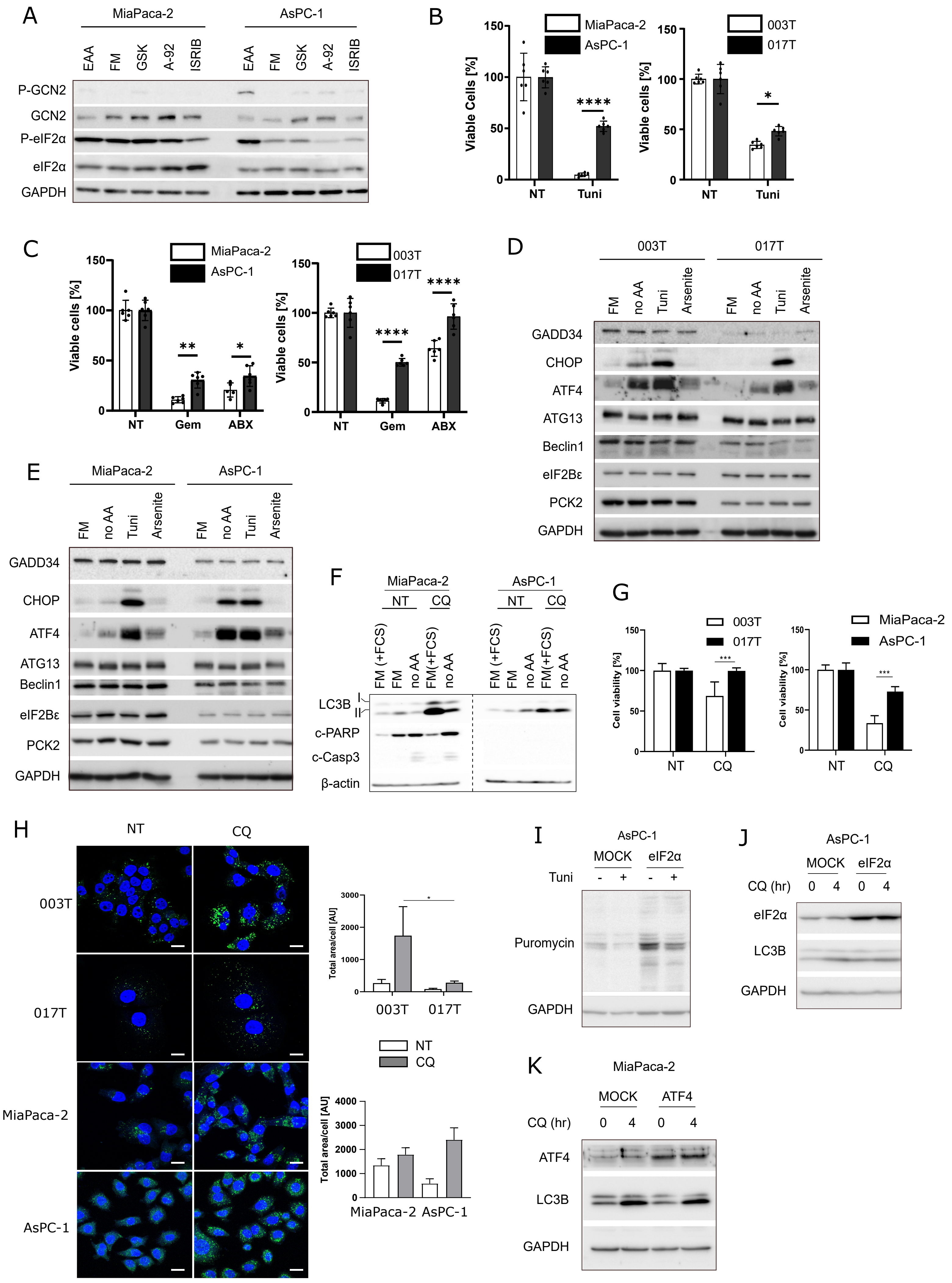

A
MiaPaca-2
AsPC-1
EAA
FM
GSK
A-92
ISRIB
P-GCN2
GCN2
P-eIF2α
eIF2α
GAPDH
B
MiaPaca-2
AsPC-1
003T
017T
Viable Cells [%]
Viable cells [%]
NT
Tuni
C
Gem
ABX
D
003T
017T
no AA
Arsenite
GADD34
CHOP
ATF4
ATG13
Beclin1
eIF2Bε
PCK2
E
F
NT
CQ
FM (+FCS)
FM(+FCS)
LC3B
c-PARP
c-Casp3
β-actin
G
Cell viability [%]
H
Total area/cell [AU]
I
MOCK
Tuni
Puromycin
J
CQ (hr)
LC3B
K

Figure S3

A) Western blot analysis of cells cultured in full media without serum (FM), treated or not with 50nM GSK, 1μM A-92 or 500nM ISRIB for 6 hrs, or in media with only essential AA (EAA) for 6hours. B-C) Viable cell quantification measured in 96 well-plate using IncuCyte imaging and PI, upon treatment with (B) 8μg/ml Tunicamycin (Tuni) or (C) with 5 μM Abraxane (ABX) or 100 μM Gemcitabine (GEM) for 48 hrs in full media. Data are presented as mean ± SD (n = 5-6). D-E) Western blot analysis of (D) 03T and 017T cells or (E) MiaPaca-2 and AsPC-1 cells cultured in full media without serum (FM) or without amino acids (no AA), or treated with 500μM Arsenite for 1 hour or 8μg/ml Tunicamycin (Tuni) for 6 hours. F) Western blot analysis of cells cultured in full media with (FM+FCS) or without serum (FM) or media without amino acids (no AA), or treated with 50 μM CQ in FM or no AA for 24 hrs. G) MTT assay with cells treated or not with 50 μM CQ for 48 hrs in full media. Data are presented as mean ± SD (n = 3). H) Immunofluorescence of LC3 in cells treated or not with 50 μM Chloroquine (CQ) for 4 hrs in full media. Total Area of LC3 per cell (right) was quantified using ImageJ. I-J) Western blot of extracts from AsPC-1 overexpressing eIF2α treated with 8 μg/mL Tunicamycin (Tuni) for 1 hr, followed by puromycin incorporation (I) or with 50 μM Chloroquine for 4 hrs (J). K) Western blot of extract from MiaPaca-2 overexpressing ATF4 treated with 50 μM Chloroquine for 4 hrs. P-values were calculated using 2-way ANOVA (*P < 0.05; **P < 0.01; ***P < 0.001; ****P < 0.0001).

Figure S4

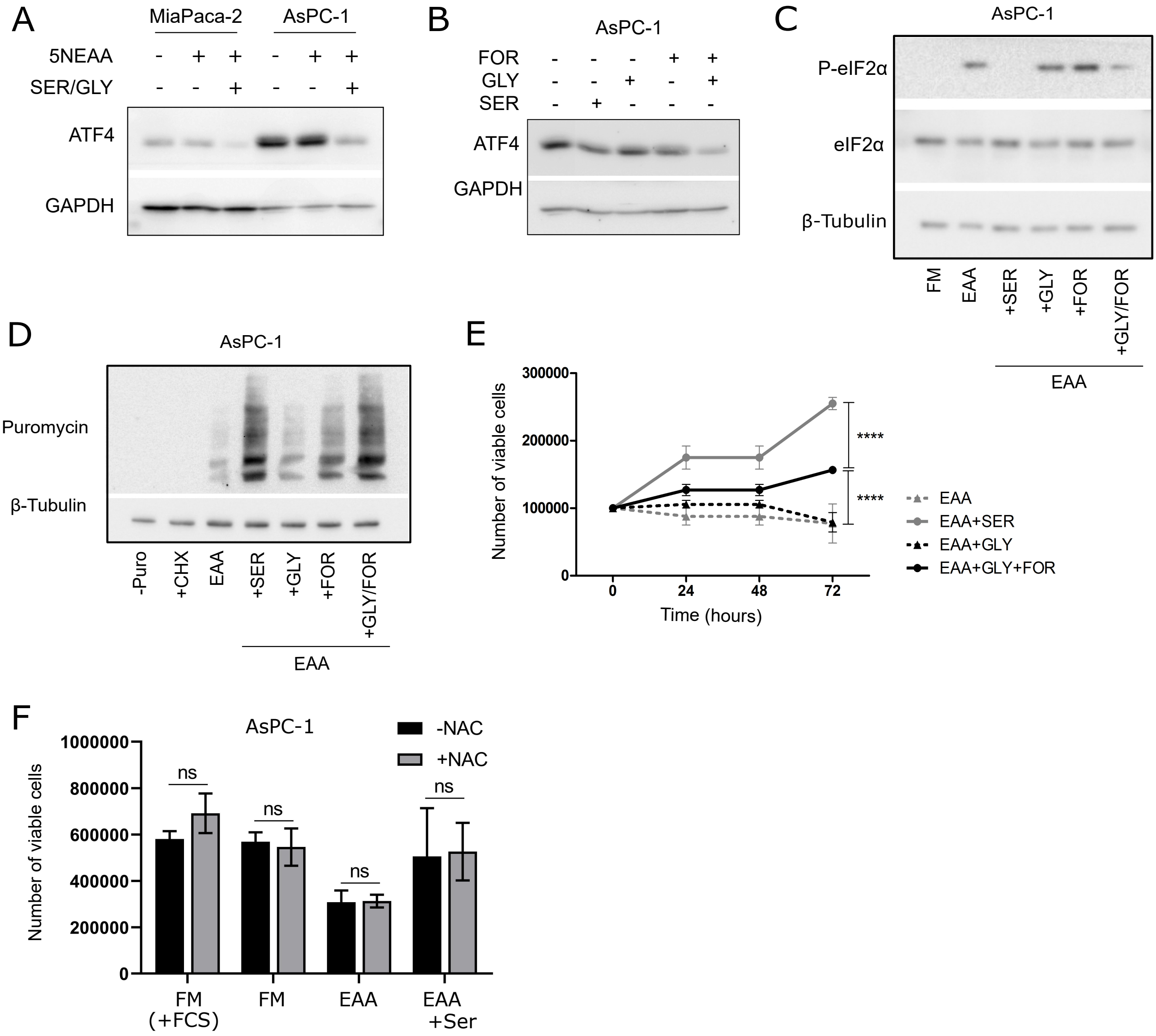
A
MiaPaca-2
AsPC-1
5NEAA - + + - + +
SER/GLY - - + - - +
ATF4
GAPDH
B
AsPC-1
FOR - - - + +
GLY - - + - +
SER - + - - -
ATF4
GAPDH
C
AsPC-1
P-eIF2α
eIF2α
β-Tubulin
FM
EAA
+SER
+GLY
+FOR
+GLY/FOR
EAA
D
AsPC-1
Puromycin
β-Tubulin
-Puro
+CHX
EAA
+SER
+GLY
+FOR
+GLY/FOR
EAA
E
Number of viable cells
300000
200000
100000
0
0
24
48
72
Time (hours)
****
****
EAA
EAA+SER
EAA+GLY
EAA+GLY+FOR
F
AsPC-1
-NAC
+NAC
Number of viable cells
1000000
800000
600000
400000
200000
0
ns
ns
ns
ns
FM (+FCS)
FM
EAA
EAA +Ser

Figure S4

A) Western blot of indicated proteins of MiaPaca-2 and AsPC-1 cells in media containing only EAA (MEM) supplemented with 100 μM proline, glutamate, asparagine, aspartate, and alanine (5NEAA) and with serine and glycine (SER/GLY) for 4 hrs B-E) AsPC-1 cells were grown in MEM (EAA) supplemented with 100 μM of serine (SER) or glycine (GLY), 500 μM of formate (FOR) or with both glycine and formate. B-D) Western blot of indicated proteins (B-C) and puromycin incorporation (D) after 24 hrs. E) Proliferation assay of AsPC-1 cells cultured in the indicated medium. Data are presented as mean ± SD (n = 3). F) AsPC-1 cell count after 72 hrs in the indicated medium with or without 1 mM NAC. Data are presented as mean ± SD (n = 3). P-values were calculated using 2-way ANOVA (*P < 0.05; **P < 0.01; ***P < 0.001; ****P < 0.0001).

Figure S5

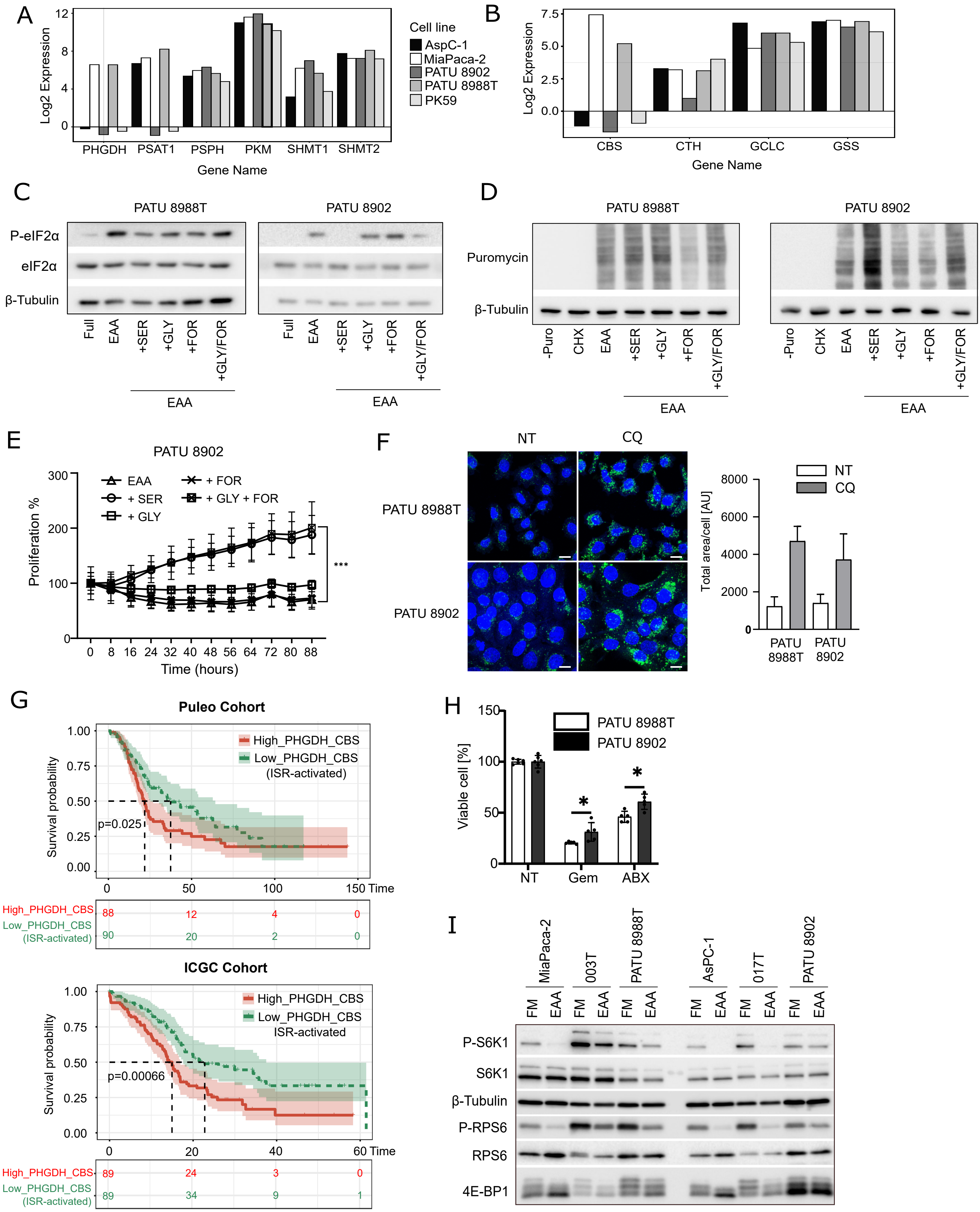

A
Log2 Expression
Gene Name
PHGDH
PSAT1
PSPH
PKM
SHMT1
SHMT2
Cell line
AspC-1
MiaPaca-2
PATU 8902
PATU 8988T
PK59
B
Log2 Expression
Gene Name
CBS
CTH
GCLC
GSS
C
PATU 8988T
PATU 8902
P-eIF2α
eIF2α
β-Tubulin
Full
EAA
+SER
+GLY
+FOR
+GLY/FOR
EAA
D
PATU 8988T
PATU 8902
Puromycin
β-Tubulin
-Puro
CHX
EAA
+SER
+GLY
+FOR
+GLY/FOR
EAA
E
PATU 8902
Proliferation %
EAA
+ SER
+ GLY
+ FOR
+ GLY + FOR
***
Time (hours)
F
NT
CQ
PATU 8988T
PATU 8902
Total area/cell [AU]
NT
CQ
PATU 8988T
PATU 8902
G
Puleo Cohort
Survival probability
High_PHGDH_CBS
Low_PHGDH_CBS
(ISR-activated)
p=0.025
Time
High_PHGDH_CBS 88 12 4 0
Low_PHGDH_CBS (ISR-activated) 90 20 2 0
ICGC Cohort
Survival probability
High_PHGDH_CBS
Low_PHGDH_CBS
ISR-activated
p=0.00066
Time
High_PHGDH_CBS 89 24 3 0
Low_PHGDH_CBS (ISR-activated) 89 34 9 1
H
Viable cell [%]
PATU 8988T
PATU 8902
NT
Gem
ABX
*
I
MiaPaca-2
003T
PATU 8988T
AsPC-1
017T
PATU 8902
FM
EAA
P-S6K1
S6K1
β-Tubulin
P-RPS6
RPS6
4E-BP1

Figure S5

A-B) Relative expression of indicated genes for SSP (A) and TSS (B) enzymes for AspC-1, MiaPaca-2, PATU 8902, PATU 8988T and PK59 PDA cell lines based CCLE RNAseq dataset. C-E) PATU 8902 and PATU 8988T cells were grown in MEM (EAA) supplemented with 100 μM of serine (SER) or glycine (GLY), 500 μM of formate (FOR) or with both glycine and formate. C-D) Western blot of indicated proteins (C) and puromycin incorporation (D) after 24 hrs. E) Cell confluence measured in 96 well-plate using IncuCyte live-cell imaging in the same conditions for 88hrs. P-value was calculated using unpaired t-test (***P < 0.001). F) Immunofluorescence of LC3 in cells treated or not with 50 μM Chloroquine (CQ) for 4 hrs in full media. Total area of LC3 per cell was quantified using ImageJ. G) Survival analysis of public PDAC cohorts exploring the impact of PHGDH and CBS expression on patient survival. Top and bottom 33% quantiles of PHGDH and CBS average expression was compared with overall survival for the Puleo Cohort on the left et the ICGC cohort on the right. H) Viable cell quantification was measured in 96 well-plates using IncuCyte imaging and PI, upon treatment with 5 μM Abraxane (ABX) or 100 μM Gemcitabine (GEM) for 48 hrs in full media. Data are presented as mean ± SD (n = 5-6). P-values were calculated using 2-way ANOVA (*P < 0.05).I) Western Blot analysis of cells cultured in full media (FM) or in media lacking non- essential AA (EAA) for 6hours.

Figure S6

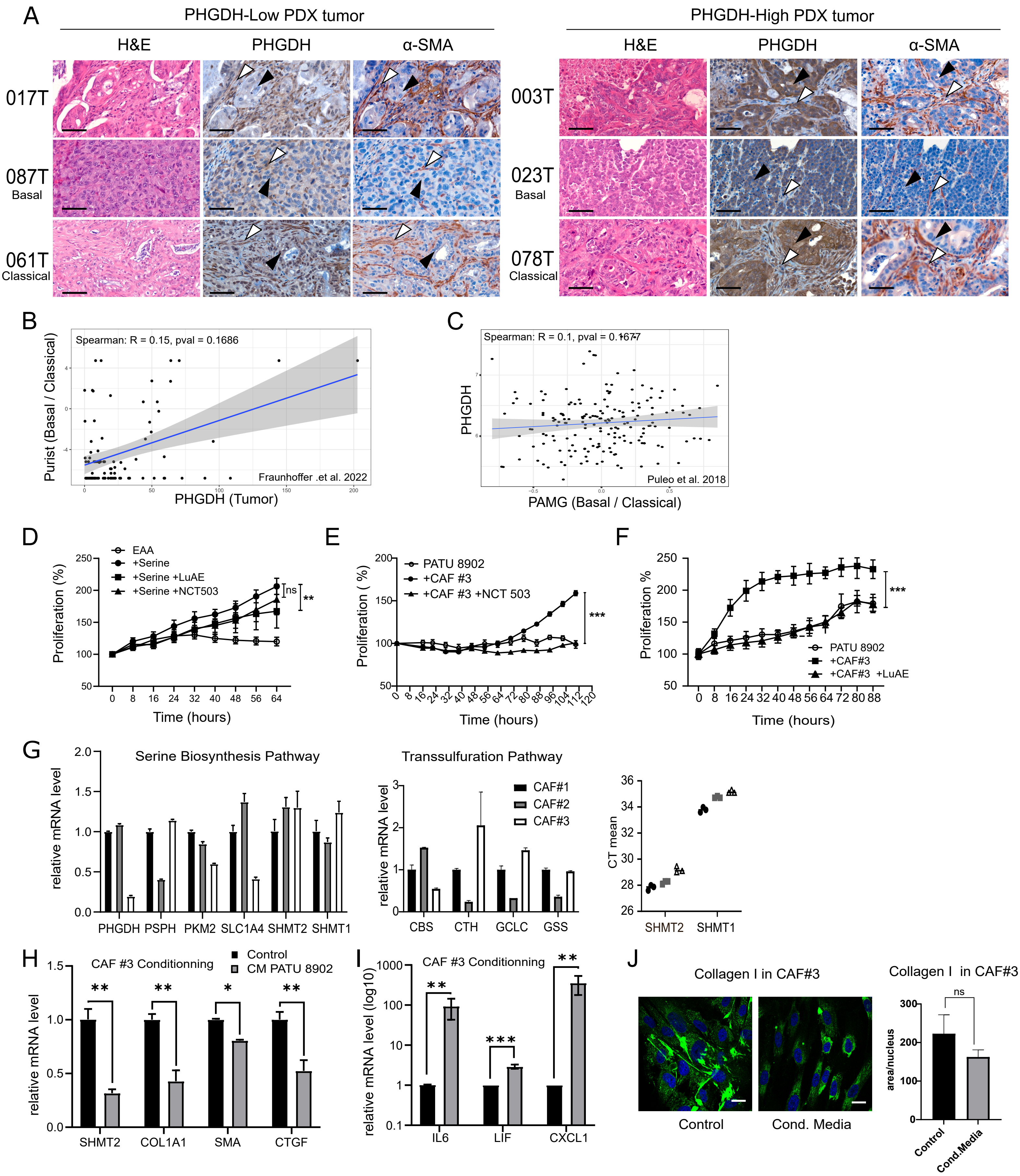

A
PHGDH-Low PDX tumor
PHGDH-High PDX tumor
H&E
PHGDH
α-SMA
017T
087T
Basal
061T
Classical
003T
023T
Basal
078T
Classical
B
Spearman: R = 0.15, pval = 0.1686
Purist (Basal / Classical)
PHGDH (Tumor)
Fraunhoffer .et al. 2022
C
Spearman: R = 0.1, pval = 0.1677
PHGDH
PAMG (Basal / Classical)
Puleo et al. 2018
D
EAA
+Serine
+Serine +LuAE
+Serine +NCT503
Proliferation (%)
Time (hours)
ns
**
E
PATU 8902
+CAF #3
+CAF #3 +NCT 503
Proliferation (%)
Time (hours)
***
F
PATU 8902
+CAF#3
+CAF#3 +LuAE
Proliferation %
Time (hours)
***
G
Serine Biosynthesis Pathway
relative mRNA level
PHGDH
PSPH
PKM2
SLC1A4
SHMT2
SHMT1
Transsulfuration Pathway
CAF#1
CAF#2
CAF#3
CBS
CTH
GCLC
GSS
CT mean
SHMT2
SHMT1
H
CAF #3 Conditionning
Control
CM PATU 8902
relative mRNA level
SHMT2
COL1A1
SMA
CTGF
I
CAF #3 Conditionning
relative mRNA level (log10)
IL6
LIF
CXCL1
J
Collagen I in CAF#3
Control
Cond. Media
Collagen I in CAF#3
area/nucleus
ns
Control
Cond.Media

Figure S6

A) Representative hematoxylin/eosin (H&E) and immunohistochemistry (IHC) stainings of PHGDH and αSMA on slices from PDX expressing low or high levels of PHGDH in the tumor compartment. Basal and Classical indicated tumor cell differentiation status, scale bar 50µm. Black arrows: Cancer cells, white arrows: stromal PHGDH staining in CAFs. B) Correlation plot of PHGDH expression in tumor cells with differentiation status (Purist) using an extended PDX cohort.[8] C) Correlation plot of PHGDH expression with differentiation status (PAMG score) using patient expression data from the Puleo Cohort.[6] D-F) Proliferation of GFP-tagged PATU 8902 alone (D) or in co-culture (E-F) with CAF #3 in the presence of NCT 503 or LuAE 00527 (LuAE). G) Relative expression of indicated genes for SBP (left) and TSS (right) enzymes for CAF #1-3 and mean CT values of SHMT1 and SHMT2 genes for CAF #1-3. H-J) CAF #3 were cultured for 24h with PATU 8902 conditioned-media (CM PATU 8902) H-I) Abundance of SHMT2, COL1A1, SMA, CTGF, IL6, LIF and CXCL1 mRNA quantified by RT-qPCR. J) Collagen expression measured by immunofluorescence. Mean fluorescence of 5 fields is shown.
